# Supplementary material for: Iterative evaluation of mobile computer-assisted digital chest x-ray screening for TB improves efficiency, yield, and outcomes in Nigeria
Source: PLOS Glob Public Health. 2024 Jan 17;4(1):e0002018. doi: 10.1371/journal.pgph.0002018 (PMC10793917; doi:10.1371/journal.pgph.0002018)
Supplement: S1 File — (PDF) [file pgph.0002018.s001.pdf]

Active Case Finding in Ogun & Nasarawa Using Mobile Chest X-ray, CAD4TB and GenXpert MTB/RIF

Planning Workshop Report

Abuja

September 19-23, 2016

Contents

Acknowledgments 6
EXECUTIVE SUMMARY 6
Introduction 7
Discussing & Defining our Approach 7
Workshop Agenda : 8
Use of Chest X-ray and Xpert for Mobile Screening in CTB 8
Matrix Teams 17
Overview of the workshop 18
Does the screening need to be targeted? or is it just any person living in a high burden community? 19
WHO Key principles of systematic screening for active TB 19
Seven recommendations on prioritizing risk groups for screening 20
Health Seeking Behavior 21
POTENTIAL SITES OF SCREENING 22
Triage in Health Care Settings- groups of interst: 22
Workplace Wellness: groups to consider 22
Urban slums- sites to consider 22
Considerations & Priorities 22
Chest X-ray: Frequently Asked Questions (F.A.Q.) 23
Why is chest x-ray a better screening tool than TB symptoms for active case finding? 23
What would be the disadvantages to simply using Xpert with traditional symptom screening (cough 2 wks) with a smaller vehicle that is only an xpert lab? 23
Why is symptom screening in the community hard? 25
How cost effective is CXR/GXP trucks screening relative to other interventions? 25
What is CAD4TB software and why is it helpful? 27
How accurate is the new version of CAD4TB that we will use? 28
Will we do a CAD4TB dose-finding exercise – a sort of diagnostic accuracy study? 30
Why would we choose to use X-ray interpretation software instead of hiring radiologists to read the X-rays? 30
COSTING OF CAD4TB: Is it worth it? 30
Leverage Predictive value of CAD4TB for future TB? 31
Experiences with mobile CXR in the Nigeria Prevalence Survey 31
What will the DAILY TB yield be from CXR/GXP? 31
Brainstorm on the cxr/xpert vehicles 32
How durable will the CXR/GXP trucks and equipment be? 32
What is required? 32
What type of facility construction is preferred? 32

ELECTRICAL Power provision 33
Truck specifications 33
X-ray equipment/Software 33
Interior Design of the truck 34
Equipment on board the truck 34
Lab-specific equipment & consumables 34
Forecasting for Commodities 34
Data management needs 35
Operations: 35
Security 35
engagement 35
Human Resources … 1 35
Linkage to treatment and care 36
Quality Sputum Collection 37
TB Screening PLHIV 38
CXR screening in PLHIV requires add'l skill 38
Can TB symptoms screens be improved so they can work better in ACF? 39
Sample TB Screening Scoring 39
Mid-Upper Arm Circumference 39
ANEMIA as predictor of TB in PLHIV 39
Visual inspection for Oral candida? 39
Nasarawa state 40
Project Areas 40
Main Risk Groups in Nasarawa State 40
Current distribution of TB burden in the LGAs 44
Main reasons for Gap in TB case detection 44
Main reasons for Gap in TB case detection 44
Programme related factors: 44
Community Leaders 45
NASARAWA LINKAGE OF DSTB, DRTB AND HIV CLIENTS TO SERVICE CENTERS 46
CHALLENGES TO TX INITIATION/ADHERENCE 46
Unclaimed results 46
Drug stock out 46
HTC AND ART 47
Is the TB treatment infrastructure sufficient? 47
Key populations and Potential Screening sites in Ogun State 49
Urban slums 49
Football stadiums 50

High Risk Occupations 51
Transport Centers- bus depots 52
Centers for Vulnerable Youth –remand ctrs 52
HIV clinics 53
HIV prevalence Ogun state 53
Private and mission health facilities 54
Ogun Team Priority LGAs (2015 est. population) 54
Prioritized Risk groups 54
Diabetes Clinics 55
Population of identified Risk groups- Ogun 56
Reasons for Gaps in Case Detection in Ogun 56
Community Level 56
Facility Level 56
Main Partners for Key populations in Ogun 57
Algorithm 57
ABNORMAL FINDINGS (NOT TB) 57
MATCHING THE SCREENING SITE TO TREATMENT SITE 57
M&E Framework 58
Defined indicators to monitor: 58
WORKSHOP RECOMMENDATIONS 59
APPENDICES 60
MOBILE CXR SCREENING DAILY SUMMARY 60
DRAFT QUESTIONNAIRE FOR PERSONS WITH GXP+ TB RESULTS 63
Energizer exercise – positioning ourselves for A C F planning 70
Participants list 71

Acknowledgments

This workshop benefitted from the strong engagement and support of, Dr Festus Olukayode Soyinka and Dr Aboki Danjuma STBLCOs for Ogun and Nasarawa State. Our work was greatly enhanced by the wisdom of the community activists and organizers who provided thoughtful insights to enhance the viability of the plans as well as used their social networks to get hard to find data to create the plans. The contributions of Dr Emperor Ubochioma (NTP) and our WHO colleagues brought the focus from local to national, and helped leverage the rich experience of the Nigerian Prevalence survey.

We were rely heavily upon the keen insights of KNCV staff - particularly Rupert Enugu, Nkem Chukwueme, Chidubem Ogbudebe, Olusola Adejumo, Peter Nwakire, .Austin Ihessie, and Gideon Zephaniah to make the work efficient and data-driven.

The fortunate coincidence of a simultaneous mission by Petra de Haas and Jerod Scholten allowed key contributions to the laboratory component of the planning. The enthusiasm of the USAID mission for the success of this work is also a source of motivation for us all. Lastly, we are indebted to Dr. Mustapha Gidado and Johan Verhoef for continuing counsel and guidance on all matters large and small.

This report is made possible by the generous support of the American people through the United States Agency for International Development (USAID). The Global Health Bureau, Office of Health, Infectious Disease and Nutrition (HIDN), US Agency for International Development, financially supports this report through Challenge TB under the terms of Agreement No. AID-OAA-A-14-00029.

The contents are the responsibility of Challenge TB and do not necessarily reflect the views of USAID or the United States Government.

EXECUTIVE SUMMARY

The ive-day workshop was implemented primarily via state-level group work, presentations by a topic experts, and thoughtful consideration of the rich experiences of the participants – many of whom brought years of community engagement and programmatic wisdom. The participants represented the State TB program, community based organizations, WHO, national TB program, donors, clinicians, epidemiologists, and TB advocacy community. Much of the conventional wisdom of TB case finding was questioned and participants engaged critically with the newer evidence regarding household symptom and smear microscopy-based ACF.

The tangible achievements of the workshop include:

1. Consensus was reached on the type of vehicle, the design, the needs, and specifications
2. Consensus reached on the target groups, methods of recruitment

3. Estimates of the magnitude of the groups at specific locations were made, and expected yields were calculated –taking into account rates of participation, drop out, pre-treatment loss to follow up
4. A decision was made to enroll those without TB -but with x-ray abnormalities – in a SMS follow-up cohort for re-testing
5. Screening and testing algorithms for each group were determined
6. M&E forms were developed
7. A choice for CAD4TB instead of human reader was made, even though we do not know the accuracy of the newest version of the tool

This report is a compilation of rapporteurs' notes and commentaries on the proceedings to enhance the institutional memory and ensure that none of the generous and thoughtful inputs of the participants are lost. The notes have been only minimally edited.

Introduction

Evidence-based active case finding to identify the missing cases in Nigeria is the top priority of the Nigerian TB program and is aligned with the goals of Challenge of TB and GFATM. ACF can be very useful, but if poorly targeted or executed, it can be a costly and even harmful exercise. Active case finding using mobile Chest-x-ray/Xpert vehicles will be implemented in Ogun and Nasarawa states in APA2.

The workshop is a broad stakeholder consultation to define the target populations, screening algorithm, treatment initiation, and follow up procedures. The consultant provided the team with evidence-based options so that the pros and cons of different operational choices are clear. The 7-step process for this workshop was derived from the WHO Operational Guidance on Screening (June, 2015) which is an inclusive, sequential approach to reaching decisions on what, where, when and how to do effective ACF.

Discussing & Defining our Approach

The ACF pilot in Ogun and Nasarawa will be conducted in an operational research framework to ensure timely information on the cost-effectiveness, yield, feasibility, and acceptability.

CTB Nigeria will ensure an adequate M&E envelope is in place to provide data for decision making on scale up. This workshop responds to needs identified in the National Epi Assessment and priorities outlined in Nigeria's national strategic plan.

Workshop Agenda :

Use of Chest X-ray and Xpert for Mobile Screening in CTB

Day 1: Monday September 19

Themes- Principles of Active Case Finding – Strategic Choices and Prioritization

		• OBJECTIVES	WHOM	LENGTH
1.	**Overview of the workshop**	• What is our mode of working together?<br>• What are we trying to accomplish this week?<br>• Who are we?	Gidado	Pres =30 min<br>Discussion 30 min
2.	**Introduction to ACF-International Framework**	• Positioning Exercise<br>• WHO Screening Principles	Ellen<br>Rupert	Exercise=30 min<br>Pres=30 min
3.	**DISCUSSION:**<br>**Why ACF?**	• What are the challenges with health seeking behavior?<br>• What are the challenges with public sector provision of Curative TB services?<br>• Who are we currently serving?<br>• What barriers prevent access to diagnostic and treatment services among the high risk groups?	Ellen	Pres=20 min<br>Discussion= 30 min
4.	**Prioritization & Sites for Screening**	• What do we want to achieve with ACF?<br>• What are the potential sites of screening?<br>• How can we create a mix of sites and communities that maximizes the yield?	Rupert	Pres+ 20 min<br>(Facilitation)
5.	**Why Chest x ray t**	• How are chest X-rays used for TB screening and diagnosis?<br>• Q&A -discussion	Ellen	Pres= 20 min<br>Discussion 45 min

6.	TB PREVALENCE	• What do we know about TB prevalence in Nigeria in Specific Groups?<br>• How do we find a case-mix that will make a difference in CNR?<br>•	WHO	30 min pres<br>30 discussion
7.	Preparation for break out sessions tomorrow	•	Rupert	20 minutes

Day 2: Tuesday September 20

Themes- State Specific Strategy Development

:

8.	Overview of ACF in Nigeria	• What were the outcomes of previous systematic screening initiatives in different risk groups?<br>• What can we learn from these experiences?	Rupert/Chidubem	30 mini
9.	CAD4TB software	• How does CAD4TB software work?<br>• Is it appropriate for this exercise?	Ellen	30 min
10.	Mobile Xpert Laboratories	• What are the opportunities and challenges presented by Mobile Xpert labs?<br>• What do we need to keep in mind in using a Mobile Xpert lab?<br>• What about electricity?<br>• What about infection control?<br>• Regulating temperature?<br>• Sample preparation<br>• Water issues<br>• Can you do sputum pooling in a mobile lab??<br>• Can you do stool ?	Petra	30 min present<br>20 min Q & A
11.	TB awareness and health seeking	• What is the level of knowledge about TB and TB care in the targeted community?<br>• What are the main reasons for delays in seeking health care among the targeted community?	Nkem	20 min pres<br>• Discussion 20 min

Afternoon: Break out into 2 state teams for Situational Assessment

12.	TB burden; Relevant Key Populations	• Which are the main risk groups in this state?<br>• What is the size of each group?<br>• Are they clustered in groups of 200 or more?<br>• What is the current distribution of the TB burden in this setting (in terms of notification, prevalence and mortality), and specifically for different subpopulations or risk groups?	Nkem (Ogun)<br>Rupert (Nasarawa)	20 min pres<br>Discussion 20 min
13.	size and distribution of gaps in case detection	• What is the current gap in case detection, and what are the specific causes of delays in diagnosis?<br>• What are the main reasons for gaps remaining in case detection?<br>• How does the state's Age and Sex disaggregated case notification match with expected burden?	Dr. Aboki Danjuma Maku (Nasarawa)<br>Dr. F.O. Soyinka (Ogun)	• 30 min
14.	Collaboration with others	• Who are the main partners in each setting who need to be engaged for the activity to be a success?	Rupert/Nkem (facilitators)	30 min
15.	Potential sites of Screening	• How large is the PLHIV cohort in care?<br>• Where/how can poor adult men be screened?<br>• How many people of high risk can be screened in a year (45 weeks)?<br>• How can sites be combined to arrive at 1000/wk or 45,000 ppl screened in 45 /wks	Dr. Aboki Danjuma Maku (Nasarawa)<br>Dr. F.O. Soyinka (Ogun)	30 min presentation<br>20 Q&A
16.	Is the TB treatment infrastructure sufficient?	• Is DOTS coverage per LGA adequate to handle new ACF cases?<br>• What MDR-TB tx gaps are there?<br>• How can these be addressed?	Chidubem	10 min pres<br>30 min discussion
17.	SYNTHESIS	• PREPARE "WHO, WHERE WHEN" presentation	Dr. Aboki Danjuma Maku (Nasarawa)<br>Dr. F.O. Soyinka (Ogun)	60 min

Day 3: Wednesday September 21

Themes- Clinical and Epidemiological Decisions

First hour:

18.	Feedback from the break out groups from yesterday	• Nasarawa – Who? Where? When?<br>• Ogun: Who? Where? When? How?	Dr. Aboki Danjuma Maku (Nasarawa)<br>Dr. F.O. Soyinka (Ogun)	90 min each

Clinical Team

19.	Adapting the process for PLHIV	• What logistical, algorithmic, adaptations are needed for screening in ART clinics?	Ellen	20 min pres<br>Discussion 20 min
20.	Chest x ray technology in the prevalence survey	• Nigerian nuclear radiation requirements<br>• How will they be trained?<br>• What will the Supervision structure be?<br>• Ensuring radiation and biosafety?<br>• Managing pregnant women<br>• What unique benefits and risks need to be planned for?	Rupert	20 min pres<br>Discussion 20 min
21.	RIF+ patient management	• if the Xpert MTB/RIF assay detects drug resistance, How to link to care, culture testing, and drug-susceptibility testing and for programmatic management of drug-resistant TB?	Rupert	20 min pres<br>Discussion 20 min
22.	Management of CXR abnormal Xpert negative	• Do we want to track the people who are Xpert negative, but CXR positive to see if they develop TB within 12 months?<br>• Diagnosis of clinical TB in the field??	Rupert	20 min pres<br>Discussion 20 min

Epidemiology & M&E teams

23.	WHO M&E of Screening	• What do we want to measure?<br>• Looking at the cascade	Chidubem	20 min pres<br>Discussion 20 min
24.	Following up a cohort of CXR abnormal	• How to track the ABN CXR cohort to capture the secondary cases?	Ellen	20 min pres<br>Discussion 20 min
25.	M&E	• What do we need to know about denominators?<br>• What do we need to know about presumptive clients?<br>• What do we need to know about cases?<br>• What about clinical cases?<br>• Do we want to follow up the ACF cohort to measure the treatment success rate ? an/ord the rate of overall loss-to-follow-up ?<br>• What New forms or registers are required?<br>• Collecting the minimum amount of data?	Chidubem	30 min pres<br>Discussion 20 min
26.	Review of the forms	• Screening summary form, presumptive register, post-test form	Chidubem	20 min pres<br>Discussion 20 min
27.	Cost–effectiveness and cost–benefit ratios	• What is a Cost effectiveness frontier?<br>• How do we know if our plan is cost effective?<br>• ACF reduces Patient costs	Ellen	20 min pres<br>Discussion 20 min

Day 4: Thursday, September 22

Themes- Operational and Community Mobilization

Feedback from the break out groups from yesterday

28.	CLINICAL	• PRESENTATION	TBD	20 min pres<br>Discussion 20 min
29.	M&E	• PRESENTATION	TBD	20 min pres<br>Discussion 20 min

Logistics and Operations Team

30.	human resources	• which cadre(s) of staff will be involved?<br>• What are their terms of reference, workload ?<br>• Security?	Ineke? Johan	30 min discussion<br>Discussion 20 min
31.	Forecasting for Commodities & Space for mobile Xpert	• Xpert cartridge forecasting<br>• Sputum cups, lab consumables<br>• CAD4TB forecasting<br>• Electricity,<br>• Petrol<br>• Data management needs<br>• Refrigeration needs (size)	Petra/Peter/Jerod	90 minutes discussion workshop
32.	BRAINSTORM on equipment	• What type of facility construction is preferred?<br>o Container based TB screening facility or<br>o rigid body built onto truck chassis?<br>• Type of equipment needed<br>o Portable or fixed CXR?<br>o How many modules of Xpert?<br>• Solar powered or not?	Johan	45 min facilitated discussion
33.	Operations	• How to manage the batteries, generators, charging?<br>• Reducing vibrations and electrical fluctuations in the field<br>• Storing and retrieving digital CXR images	Johan /peter	45 min facilitated discussion

Communication & Social Mobilization

34.	**Synergies?**	• Should we offer diabetes, hypertension, eye exams or other screenings?<br>• Should we offer HIV testing? When ? how?<br>• What kinds of referrals will we do?	Rupert	30 min discussion
35.	**Social Mobilization and Recruitment**	• What special considerations are needed for the chosen groups?<br>• Review the mapping of the screening and outreach sites?<br>• Consenting ?<br>• Current radio jingle? Is it appropriate?<br>• How to leverage the call-in line?	Nkem	90 min discussion
36.	**Following up on CXR abnormal**	• How can we motivate people for re-testing 6 to 12 months after the initial screen?		

Day 5 Putting it All Together

Themes-

Feedback from the break out groups from yesterday

37.	**Feedback from Social Mobilization**	• Teachback		30 min pres
38.	**Logistics & Operations**	• Teach back		30 min pres

State Teams

39.	**Linkage to care**	• How to ensure treatment initiation? How can we ensure low rate of initial loss to follow-up? What are the specific challenges to initiating and adhering to treatment in each group?<br>• Matching the screening sites to treatment sites<br>• Planning for Industrial actions	Rupert/Nkem/Chidubem/ Chidubem	2 hours
40.	**Linkage to TB drugs**	• Are there regular and reliable supplies of anti-TB medicines are available? Is there the capacity to treat the anticipated rise in cases of drug-susceptible as well as drug-resistant cases among adults ?	Dr. Aboki Danjuma Maku (Nasarawa)<br>Dr. F.O. Soyinka (Ogun)	
41.	**Team work**	• Drafting state plans – targets, case-mix,	Dr. Aboki Danjuma Maku (Nasarawa)<br>Dr. F.O. Soyinka (Ogun)	1 hours
42.	**Final presentations**	• Final presentations	Dr. Aboki Danjuma Maku (Nasarawa)<br>Dr. F.O. Soyinka (Ogun)	1 hour
43.	**Closing and appreciations**	• Closing and appreciations	Gidado	10 min

Afternoon: Integration of all the individual work into final plans & Presentation of the final plans by state teams

Matrix Teams

Civil Society Team	Epidemiology M&ETeam	Logistics and Operations Team	Ogun State Team	Nasarawa State Team	Clinical Team
Babagana Adams<br>Mayowa Joel<br>Dr Osakwe<br>Mrs Ibiyemi Fakande<br>Dr Emperor<br>Ubochioma<br>Chidubem Ogbudebe	Chidubem Ogbudebe<br>Gideon Zephaniah<br>Stephanie Gande<br>Ifiok Ekanim<br>Ellen Mitchell	Abdul Rasak Dikko<br>Dr Enang Oyama<br>Peter<br>Johan<br>Rupert Enogu	Dr Festus Olukayode<br>Soyinka<br>Dr Nkem<br>Chukwuemeka<br>Chidubem Ogbudebe<br>Ifiok Ekanim<br>Dr Enang Oyama<br>Dr Austin Ihesie	Dr Aboki Danjuma<br>Abdul Rasak Dikko<br>Rupert Enogu<br>Stephanie Gande<br>Gideon Zephaniah<br>Dr Emperor<br>Ubochioma<br>Dr Omoniyi Fadare<br>Dr Titilope Ogunlade	Dr Omoniyi Fadare<br>Dr Enang Oyama<br>Dr Nkem<br>Chukwuemeka<br>Rupert Enogu<br>Dr. Gidado Mustapha<br>Dr Austin Ihesie<br>Dr Titilope Ogunlade

Overview of the workshop

Total yield of *true* TB cases

Equity aspects of ACF

Accounting for variable feasibility and acceptability of ACF among different populations

Number Needed to Screen (NNS) to find 1 True case – a key metric

Cost effectiveness and cost benefit

Impact on transmission

Benefits vs. harm for the individual

Puzzles

Puzzles

Workshop Flow

Matrix Teams

<u>Day 1: Monday September 19</u>

Objectives: Principles of ACF – Ethics, Equity?, Disparity?

Qs:

Why do we innovate? How do the traditional case-finding approaches fair?

What are our values with respect to different risk groups and their relative priority?

<u>MONDAY goals & objectives</u>

Primary aim: detect active TB early?

Reducing the risk of poor treatment outcomes and the adverse social and economic consequences.

Equitable access

Reducing TB transmission

Secondary:

Rule out active disease to help identify people who are eligible for treatment of latent TB infection?

Identifying people who are at particularly high risk of developing active disease in the future and thus may require repeat testing?

<u>Day 2: Tuesday September 20</u>

Objectives: State Specific Strategy Development

Qs::

Set targets

Combine sites/groups to meet targets

State Situation Assessment

- ☐ TB epidemiology and case detection gaps
- ☐ Map risk groups and special access barriers
- ☐ Preparedness of NTP, health system, partners
- ☐ Existing regulatory and human rights frameworks

<u>Day 3: Wednesday September 21</u>

Objectives: Clinical and M&E Strategy

Qs::

- ☐ How to address specific clinical issues
- ☐ How to capture the performance & results

4. Choosing algorithms

- ☐ Accuracy and yield of the screening and diagnostic tests
- ☐ Reducing risk of false positive diagnosis
- ☐ Estimating TB prevalence in the risk groups
- ☐ Availability, feasibility, capacity for using different tests, throughput, etc
- ☐ Cost and cost-effectiveness

Day 4: Thursday, September 22

Objectives: Operational & Logistical Issues

Qs::

How many staff do we need?

How many cartridges? CAD4TB scans?

Day 4: Planning, budgeting and implementing

Planning, HR and commodity requirements, budgeting

Partner and community involvement

Resource mobilization

Day 5: Friday, September 23rd

Objectives: Linkage to care, draft plans

Qs:

- ☐ Ethical considerations (e.g. parental consent for minors etc.)
- ☐ Ensuring high quality treatment for TB patients we help to find

Does the screening need to be targeted? or is it just any person living in a high burden community?

Yes targeting of high risk groups is needed. If low risk groups are included, then CXR/GXP trucks will be unsustainably expensive.

For example, WHO notes that **healthy children 5-14 years should be excluded from TB screening** because they represent a very low risk group AND more importantly they constitute a huge proportion of the Nigerian population. So if they are included in TB screening programs (WHO advises against TB screening in children) then the intervention will probably not be justified on cost effectiveness grounds. Similarly the focus should be on adult men instead of young women of reproductive age.

WHO Key principles of systematic screening for active TB

Principle #1b

In addition, a baseline analysis should be completed in order to demonstrate that the potential benefits of screening clearly outweigh the risks of doing harm, and that the required investments in screening are reasonable in relation to the expected benefits.

Principle #2

Indiscriminate mass screening should be avoided. The prioritization of risk groups for screening should be based on assessments made for each risk group of

- ☐ the potential benefits and harms,
- ☐ the feasibility of the initiative,
- ☐ the acceptability of the approach,
- ☐ the number needed to screen,
- ☐ the cost effectiveness of screening.

Principle #3

The choice of algorithm for screening and diagnosis should be based on an assessment of the accuracy of the algorithm for each risk group considered, as well as the availability, feasibility and cost of the tests.

Principle #4

TB screening should follow established ethical principles for screening for infectious diseases, observe human rights, and be designed to minimize the risk of discomfort, pain, stigma and discrimination.

Principle #5

The TB screening approach should be developed and implemented in a way that optimizes synergies with the delivery of other health services and social services.

Principle #6

A screening strategy should be monitored and reassessed continually to inform re-prioritization of risk groups, re-adaptation of screening approaches when necessary and discontinuation of screening at an appropriate time.

Recommendations on risk groups to screen

Seven recommendations on prioritizing risk groups for screening

Seven recommendations on prioritizing risk groups for screening have been developed. The recommendations are divided into strong recommendations and conditional recommendations.

A strong recommendation is one for which the desirable effects of adhering to the recommendation are judged to clearly outweigh the undesirable effects, and for which screening is judged to be feasible, acceptable and affordable in all settings.

A conditional recommendation is one for which the desirable effects of adhering to the recommendation probably outweigh the undesirable effects but the trade-offs, cost effectiveness,

feasibility or affordability, or some combination of these, are uncertain. Reasons for uncertainty may include:
a lack of high-quality evidence to support the recommendation;
high costs or low feasibility or acceptability, or a combination of these.

Recommendation 1: Household contacts and other close contacts should be systematically screened for active TB.
Recommendation 2: People living with the human immunodeficiency virus (HIV should be systematically screened for active TB at each visit to a health facility.
Recommendation 3: Current and former workers in workplaces with silica exposure should be systematically screened for active TB.
Conditional recommendations
Recommendation 4: Systematic screening for active TB should be considered in prisons and other penitentiary institutions.
Recommendation 5: Systematic screening for active TB should be considered in people with an untreated fibrotic chest X-ray lesion.
Recommendation 6: In settings where the TB prevalence in the general population is 100/100 000 population or higher, systematic screening for active TB should be considered among people who are seeking health care or who are in health care and who belong to selected risk groups.
Recommendation 7: (a) Systematic screening for active TB may be considered for geographically defined subpopulations with extremely high levels of undetected TB (1% prevalence or higher). (b) Systematic screening for active TB may be considered also for other subpopulations that have very poor access to health care, such as people living in urban slums, homeless people, people living in remote areas with poor access to health care, and other vulnerable or marginalized groups, including some indigenous populations, migrants and refugees.

Health Seeking Behavior

Among 1,142 individuals interviewed for health-seeking behavior through Nigeria's 2012 National TB Prevalence Survey,
45% first sought care from a hospital. (National Prevalence Survey Report, 2014).
In the Nigerian National Prevalence Survey, only 26% of TB patients sought treatment at the public primary health care level. Ten percent were cared for in the private hospitals and another 6% in mission facilities (1).
In a second general health survey administered to public (n=318) and private health care workers (n=113) and community members (n=215), only 17.2% of participants reported that they would prefer to attend a government hospital to receive health services (17).
The majority selected private clinics, faith-based hospitals/clinics or others for treatment. Notably, only 35.5% of public health workers and 11.6% of community members preferred to attend a government hospital (17).
These results seem to indicate a general low confidence in public facilities, in spite of higher numbers of well-trained health staff. (Not suprising given these well-trained staff are often in the middle of an industrial action)...

POTENTIAL SITES OF SCREENING

Triage in Health Care Settings- groups of interst:

- ☐ People previously treated for TB
- ☐ People with an untreated fibrotic chest radiography lesion
- ☐ People living with HIV / People attending for HIV testing
- ☐ People with diabetes mellitus
- ☐ People who smoke / People with chronic respiratory disease
- ☐ Undernourished people
- ☐ People who have had a gastrectomy or jejunoileal bypass
- ☐ People with chronic renal failure
- ☐ People on treatments that compromise their immune system
- ☐ Elderly people
- ☐ People in mental health clinics or institutions
- ☐ General inpatients

Workplace Wellness: groups to consider

Silicosis makes an individual more susceptible to TB. Work profiles:

- ☐ Sandblasting for surface preparation.
- ☐ Crushing and drilling rock and concrete.
- ☐ Mining/tunneling; demolition work.
- ☐ Masonry and concrete work
- ☐ building and road construction and repair.
- ☐ Cement and asphalt pavement manufacturing

Urban slums- sites to consider

- ☐ Foot ball viewing centers
- ☐ Markets
- ☐ Transport hubs- mechanics' villages

Football viewing centers

105 minute matches- half time

Open markets

Bus Stops and Transport hubs

Active Case Finding

Planning for Results

ACF is a shortcut to care

Considerations & Priorities

Should we be looking for the highest number of cases at the lowest price?

Should we be trying to avert the greatest amount of transmission?
Should we be trying to save the most lives?
How expensive should it be to find a single true TB case?
US$350?
US$750?
US $<1000?

Weekly Throughput/Yield
Untargeted Screening is not endorsed by WHO unless the community prevalence is above 1% -- (that only applies in parts of Swaziland and Lesotho).

Chest X-ray: Frequently Asked Questions (F.A.Q.)

Why is chest x-ray a better screening tool than TB symptoms for active case finding?

Chest x-ray correctly identifies approximately 20-40% more people who need TB testing than other methods(1). It saves money and time by reducing the number of people who are tested unnecessarily(1).

However, inappropriate mass use of CXR screening in young people can be a problem in Nigeria and many other countries. Indeed it is not uncommon to require CXR screening for TB for school going young people as part of the admissions process (5). A recent audit of 3859 pre-admission chest radiographs of apparently health students at the University of Benin in Edo state found 99.7% were normal. Two males were diagnosed with TB, with a prevalence of 51/100,000. This screening costs approximately 7 million naira in 2010 and probably does more harm than good.

In addition to pinpointing who may have TB disease in the present, abnormal chest x-rays offer good guidance for predicting who may develop TB disease within one year(2,3). In Vietnam and Cambodia, approximately 6-8% of people with abnormal chest-x-rays (and smear negative initial results) go on to develop TB within 12 months.(2,3) Therefore chest x-ray doubles as a screening tool and an early warning system, helping in the diagnosis of TB in two ways. Few programs have tried to leverage the potential of CXr as an early warning system but establishing a follow up (re-test) mechanism may enhance the effectiveness of mobile outreach.

What would be the disadvantages to simply using Xpert with traditional symptom screening (cough 2 wks) with a smaller vehicle that is only an xpert lab?

Traditional symptoms screens (cough > 2 weeks) allows for finding 30-50% of people with TB (depending on the HIV prevalence of the screened group). So even if you follow it with a highly accurate diagnostic (Genexpert Ultra) that can capture 80-95% of TB, most of the people with TB will not be flagged for Xpert during the initial screen. Most of the TB is lost by using a poor screening

method (ie symptoms). Household screening with symptoms is a poor approach. This is clear from the modelling study by Van't Hoog et al 2013 (data below). In a general all-ages urban slum in Nigeria, the pulmonary TB prevalence is unlikely to be higher than 0.5% of the population (500/100,000). Cough for 2 weeks followed by Xpert identifies 32% of the TB, whereas CXR plus Xpert identifies between 80-90% of the TB depending on the threshold you use for investigation. Even if you expand the symptom screen to one that is more sensitive, the specificity drops so quickly (i.e. too many are flagged for Xpert testing) that it becomes too expensive and PPV approaches coin-flip.

Table 3: Comparison of Screening and Testing Algorithms

Algorithm	First screening test	Second screening test		Positive predictive value (PPV)		Negative predictive value (NPV)		% true cases detected by algorithm alone		Proportion testing negative who go on to valid Clinical Diagnosis	
				Prev = 0.5%	Prev = 1%	Prev = 0.5%	Prev = 1%	Prev = 0.5%	Prev = 1%	Prev = 0.5%	Prev = 1%
C2WK=SSM	Cough >2 weeks	Sputum smear microscopy		50	67	99	97	21	21	20	30
C2WK=GXP	Cough >2 weeks	Xpert		75	86	100	100	32	32	5	5
ANY5SYM=SSM	Any symptoms	Sputum smear microscopy		27	42	100	99	47	47	5	10
ANY5SYM=GXP	Any symptoms	Xpert		52	69	100	100	71	71	5	5
TBCXR=SSM	CXR (TB abnormal)	Sputum smear microscopy		56	72	98	97	53	53	20	40
ABCXR=SSM	CXR (any abnormal)	Sputum smear microscopy		38	55	99	98	60	60	10	20
TBCXR=GXP	CXR (TB abnormal)	Xpert		79	88	100	99	80	80	5	10
ABCXR=GXP	CXR (any abnormal)	Xpert		65	79	100	100	90	90	5	5

We can design an Implementations Science effort to compare use of only Xpert labs in some states against CXR/GXP trucks in other states. That would be a very useful exercise, but given the improvements in Version 5 of CAD5TB, I think the results would still favor CXR/GXP solutions.

Why is symptom screening in the community hard?

Symptom screening is challenging because TB symptoms are not *pathognomonic and this triggers over-referral and under-referral*. This wastes resources and can result in delegitimization of community volunteers) in the eyes of both communities and health workers. A recent review of ACF in Nigeria suggests that most community based symptom screening conducted is ineffective due to low volumes of referral(6).

How cost effective is CXR/GXP trucks screening relative to other interventions?

Cost effectiveness of CXR/GXP trucks will be a function of:

- the volume of screening done
- the pre-test probability to TB in the group selected for screening
- the life span of the equipment

If you have low volume of people screened per day, mobile trucks are indeed an extremely expensive solution. If you have high volumes, it quickly becomes cost effective. Reaching the tipping point for cost effectiveness requires the truck to be screening minimally 200 adults per day for at least 250 days per year(1).

To do some scenario testing, we assumed asset of sensitivities and specificities and costs for various tests. We included some screens that do not currently exist (e.g. hypothetical improved TB symptom score), but could be developed in the near future. For CAD4TB, we used the manufacturer's estimated sensitivity/specificity estimates (90/80), which are higher than previous estimates (86/75). We did this despite the fact that, to our knowledge, the new version has yet to be rigorously tested in a proper study.

Table 2: Accuracy and costs of Certain Screening and Diagnostic Tests

desc	sens	spec	Diagnostic cost	Operational cost
X-ray (any abnormality)	91%	75%	$ 5.00	$ 4.50
X-ray (suggestive of TB)	87%	96%	$ 5.00	$ 5.60
X-ray (after sym. screening)	90%	56%	$ 5.00	$ 4.50
CAD4TB Version 5	89%	80%	$ 5.00	$ 5.50
Cough >2wks	35%	95%	$0	$ 3.00
Any cough	51%	93%	$0	$ 3.00
Hypothetical Improved TB Symptom Score	70%	83%	$0	$ 3.00
Pooled Ultra 3 samples	78%	98%	$3.33	$ 3.00
Pooled Ultra 5 samples	75%	100%	$ 2.00	$ 3.00
Microscopy	61%	97%	$ 2.00	$ 0.50

Xpert MTB/RIF -HIV negatives	92%	98%	$10.00	$ 3.00
Clinical diagnosis	33%	94%	$0	$ 5.00

Under these conditions, CAD4TB with Ultra is on the cost effectiveness frontier and dominates any form of symptom screening followed by Xpert.

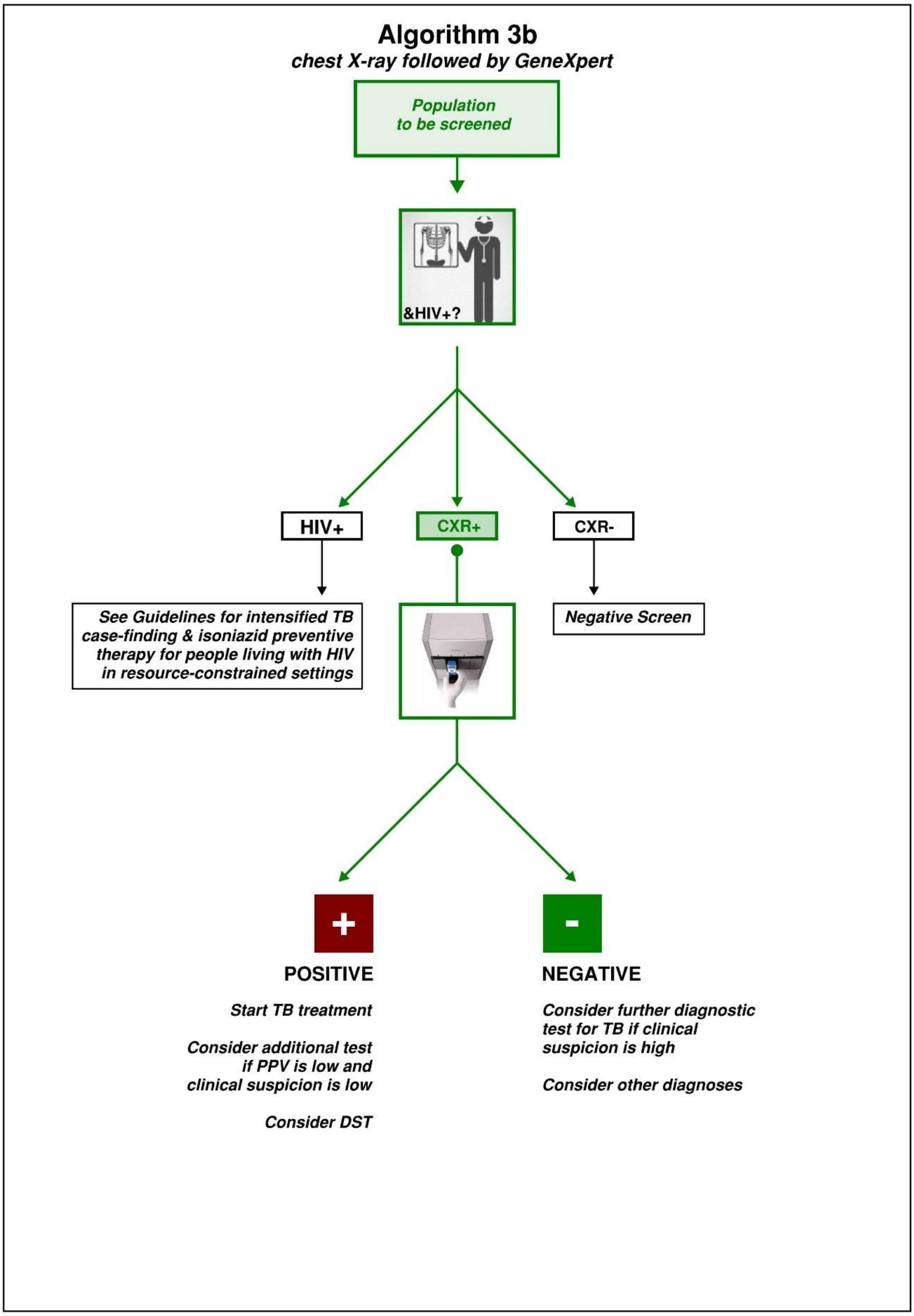

What is CAD4TB software and why is it helpful?

Computer assisted Diagnosis for Tuberculosis (CAD4TB) technology was developed in Raboud University by Bram van Ginneken in cooperation with Delft Imaging Systems(7). The software has

gone through several improvements and version 4 has a reported sensitivity of 86%, specificity 80% for pulmonary TB in HIV-negative clients(7). The area under the ROC curve of CAD4TB version 4 for the detection of culture-positive pulmonary tuberculosis was 0.84 (95% CI 0.80–0.88)(7).

CAD4TB software is European Conformity (CE) certified and CAD4TB is described in the WHO 2012 Compendium of Innovative eHealth Technologies. The software can process a digital image within one minute and therefore it can be incorporated into a busy active case finding project without undue burden on participants or staff(3).

Version 4 of the CAD4TB software has the capacity to

1. Classify normal vs. abnormal digital chest radiographs;
2. Mark suspect regions;
3. Combine shape-texture-symmetry abnormality scores;

The software "reads" a digital image and instead of a dichotomous reading (Yes/No TB), it generates a probability score for TB ranging from 0 to 100. This continuous variable permits programs to establish testing thresholds that favor either comprehensiveness or efficiency (see Table 1). This feature makes it particularly well-suited for active case finding in diverse contexts.

Table 1 Illustrative Probability Cut-Offs for v. 4 CAD4TB in Sub-Saharan Africa(7)

	Cut off value	Sensitivity 95%CI	Specificity 95%CI
Option 1	≥56	84 (79-90)	69 (62-75)
Option 2	≥37	91 (86-94)	52 (46-59)
Option 3	≥ 23	95 (91-98)	33 (27-29)

Previous versions of CAD4TB software performed as well as a human medical officer in the interpretation of CXR(8). Version 3.07 appears to offer superior accuracy to clinical officers when only TB-specific abnormalities are considered(7). However the software is currently less accurate than a trained, experienced Radiographer for detection of TB(7).

As with all screening tools, CAD4TB has higher positive predictive value with smear positive and HIV-negative TB patients(7,9). However, compared to symptom screening, tuberculin skin test, or IGRA (other TB screening tools available) it is more accurate, contributing to more strategic utilization of expensive downstream diagnostic tests(10,11). As an automated screening test coupled with Xpert, modelling studies suggest that it can reduce time to treatment, potentially interrupting transmission(8).

How accurate is the new version of CAD4TB that we will use?

We don't know.

Inventor of CAD4TB: Bram van Ginneken,
Diagnostic Image Analysis Group,
Radboud University Medical Center,
Nijmegen, The Netherlands

CAD4TB timeline
1996: Delft Imaging Systems launches first digital X-ray for TB screening; Dutch grant to develop software to analyze radiographs, they hired Bram van Ginneken
2001: Thesis and first publications on automated detection of TB from chest radiographs
2006: Delft Imaging Systems launches integrated digital screening solution
2008: Digital chest radiography units installed in Zambia and South Africa
2010: Bram van Ginneken moves to Radboud University Medical Center, starts DIAG
2011 - 2014: Four releases of CAD4TB
CAD4TB – Usage 2014

CAD4TB creates a **HEAT MAP-** 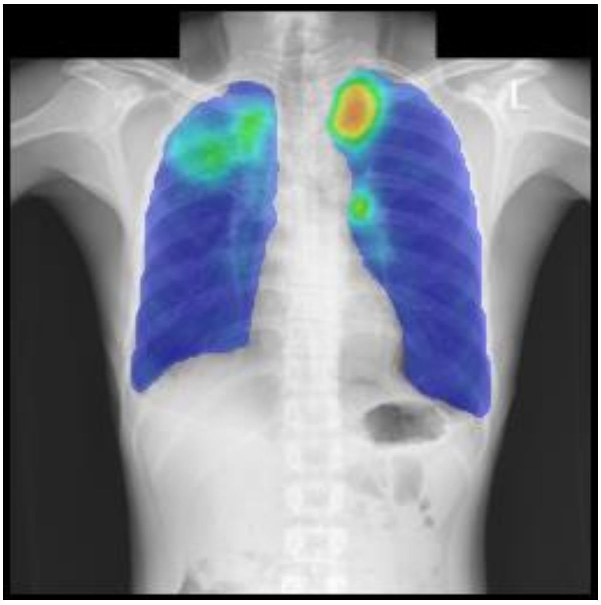

This is a printable picture that may serve as a cue-to-action for people who are TB NEGATIVE on the day of xpert testing, but who may have incipient disease and merit re-testing.

Current Studies ongoing of CAD4TB

1. Results WHO specimen bank Kanyama
2. Prison screening Tanzania
3. Andreas Steiner et al. Union conference. Reader A:Reader B:
4. Results for Palawan DetecTB (PHILIPPINES)
   Results for full data set
   Illustrative Probability Cut-Offs for v. 4 CAD4TB in Sub-Saharan Africa (High HIV)

Will we do a CAD4TB dose-finding exercise – a sort of diagnostic accuracy study?

No. We are not scientifically validating CAD4TB technology in this ACF intervention. The focus is on finding TB cases and treating them.. Instead, we will choose the best Single Cut-off (Threshold) Value for Xpert Testing and apply it based upon the best available information and science at the time of the launch. We may do an initial calibration exercise –where the first 1000 people are tested for Xpert, but this will be dependent on budget and stakeholder discussions.

Why would we choose to use X-ray interpretation software instead of hiring radiologists to read the X-rays?

In field-based mass screening (TB active case finding), Radiologists are over-qualified to do the type of crude interpretation required in high volume TB screening. TB screening is not diagnostic. It requires only a broad understanding of TB features because typically any abnormality is considered grounds for a bacteriologist test. Increasingly the task of crude- preselection (aka screening) is being automated to allow better access to persons outside of major metropolitan areas where radiology expertise is concentrated. Task shifting allows expert radiologists to focus on complex tasks(3).

Historically, TB diagnosis with chest radiography has been fraught with subjectivity(4,5). In a screening scenario the images are often of very early TB disease, which can be subtle and smear negative(6). Interpretation of the finer details of hundreds of CXR per day can be taxing on the human eye and quality of reading can decline over a workday. Therefore the removal of human variability in TB screening is seen by some as advantageous. Automation provides a standardized level of quality and high throughput which can be maintained during many hours of continuous service making it conducive for use during TB screening campaigns.

The quantitative nature of the CAD4TB likelihood score facilitates careful calibration of the intensity of the TB screen to the absorptive capacity of the lab. In other words, the threshold value for TB investigation (proportion of people sent for testing) can be adjusted in accordance with the background epidemiology of the population, the desired degree of confidence, the lab size and workflow, etc.

Comparison
Human x-ray Reader=Payment independent of productivity

COSTING OF CAD4TB: Is it worth it?

Pay $1.25-1.75 per scan for first 20,000 scans
Price drops if volume increases
Currently Sold in batches of 20,000
Recommendation: We should engage with the copyright holder to see if we can interest them in a new kind of research, which they would subsidize – looking at the predictive value of the technology.

Conclusions

Consistent improvement in CAD4TB from version 1.08, 2.09, 3.07, 4.10

- Version 4.10 requires <1min computation time on quad-core PC
- Automated reading with a performance comparable to a human expert is possible: huge logistic advantages
- CAD4TB works on X-rays from different manufacturers
- Release v4.10 HAS CE certification
- We would use v 5.0

Future CAD4TB research on-going

- Automated reading needs to be tested in prospective studies, in various settings (passive case finding, active case finding, prevalence surveys), with various populations (clinical characteristics), with various algorithms
- CAD4TB still only gives one overall score.
- Future Extensions??:
    - Differentiate TB from other lung diseases?
    - Differentiate active and old TB?
    - Quantify lesion load (and temporal change)?
    - Adapt for pediatric TB?
    - Adept software to specific use (retraining, online learning)

    Improve further and detect more signs of TB

Leverage *Predictive* value of CAD4TB for future TB?

- Immediate Yield
- Follow up Yield

Human Reader

Payment independent of productivity

CAD4TB

Experiences with mobile CXR in the Nigeria Prevalence Survey

Nigeria prevalence survey 2012

5.9% of the population was CXR Abnormal

Pay $1.75 per scan FOR cad4tb

Price drops if volume increases

Sold in batches of 20,000

Mobile CXR/Xpert

What will the DAILY TB yield be from CXR/GXP?

If we screen 250 people in urban areas per day, assuming urban prevalence of 638/100,000

CAD4tb_xPERT	1	1	0	248	1	3956.871	3957	250
T2sem_Ultra	1	0	1	248	1	970.6345	971	250
Ultra	1	2	0	246	1	4250	4250	250
T1sem_CR_ultra	0	0	1	248	2	974.7404	Inf	Inf

T1sem_ultra	1	0	1	248	1	1059.431	1059	250
Melhor_sint_ultra	1	0	1	248	1	1486.871	1487	250
CAD4TB_ultra	1	1	0	248	1	3707.413	3707	250
PoolUltra_5	1	4	0	245	1	2125	2125	250
PoolUltra_7	1	4	0	245	1	1375	1375	250
CR_GP	1	1	0	248	1	3956.871	3957	250
Custom1	2	248	0	0	0	0	0	125
Custom2	2	248	0	0	0	0	0	125

Brainstorm on the CXR/XPERT vehicles

How durable will the CXR/GXP trucks and equipment be?

The discussion around cost effectiveness is driven by how many years the initial capital investment continues to function well. Many modelling studies use 15 years as the average life span of a CXR machine and 5- years as the depreciation period (1,4).

However, real use will be a better determination of how long the tools actually work. The Minray machines used in the prevalence survey were quickly damaged by the introduction of software viruses and only 3 out of 5 were able to be subsequently deployed.

The longevity of a GeneXpert machine that is in a mobile unit may not be the same as one in a static laboratory environment with stable electricity. Having both a mobile Xpert machine and a CXR on the same electrical grid in a truck has never been attempted and is not recommended. All current should be run through separate batteries and stabilizers to prevent surges.

What is required?

2x4, 4x4 or 6x4 configuration?

Is 'poor road / off road' capability required?
Is high ground clearance required?
Theft prevention?

What type of facility construction is preferred?

Container-based TB screening facility or
Rigid body built onto truck chassis?
TB CXR and Xpert Malawi
Truck with trailer generators
CXR/Xpert South African Prisons
Zambia trucks, removable containers
Giant buses? (Thailand)

Kenya 2008 –
-X

How should access for clients into the screening compartment be ensured:
A simple ladder with railing against the open door?
A platform sliding out from under the door with a ladder & railing?
Something completely different?
Do clients need to undress in a ‘waiting room’ and is any facility needed for that?

ELECTRICAL Power provision

Xpert machines in Mobile clinic need stable electricity or high error and failure rates
Not universal; never tried before.

Battery pack with inverter for stable current (4 hour capacity)?
Batteries replenished with
Solar panels on roof? (retrofit after shipment in view of transport costs)
Link to power grid?
Link to Truck engine alternator?
Diesel-powered generator 7-8 KvA?
Permanently built-in or portable?
Ventilation + Airco needed 24/24!

Truck specifications

Is there, in view of local servicing, a preference towards a specific vehicle manufacturer (Iveco, Misubishi, Toyota, Nissan, DAF, MAN, VW…)?
Petrol or diesel engines?
8-ton truck needed (container = 7 ton)
MAN maintains factory warranty in Nigeria
Automatic gearbox or stick shift?

X-ray equipment/Software

Is there any preference towards an X-ray modality vendor? (e.g. OlDelft, Philips, Samsung, GE, OR-Technology…)
Is Delft Imaging Systems’ CAD4TB compatible with other X-ray equipment?
Is the Indian competition for CAD4TB already on the market?
Fixed or portable?
MinMax (U.S.A.) Used in Nigeria Prevalence Survey
Other points to consider…
GeneXpert to be procured separately & retrofitted?
Same for microscope (if needed)? NO MICROSCOPE
Is water tank (volume needed??) or connection to external pipe system needed for the lab space?
+…? Logistics Presentation
Systematic screening for active TB using mobile trucks
Type of truck and Container
6 wheel drive truck with high clear from the ground
A detachable container (20 feet by 8 feet)

Container-based TB screening
Hydraulic resting platform

Interior Design of the truck

Such to ensure unidirectional movement of clients – 2 doors in CXr?
Should contain the following units/cabin:
Chest x-ray cabin fortified with lead walls
With a small private corner
Film shooting cabin/unit
GeneXpert lab
Convenience if possible

Equipment on board the truck

Portable flexible x-ray (modality vendor-Ol Delft) x 1
4 or 16 modular GeneXpert machines x 2
Fixed (protected) laptop computers
Portable refrigerators
Air conditioner
Other GeneXpert accessories
GxAlert system (internet connectivity = dual capability routers)

Lab-specific equipment & consumables

Biosafety cabinet and extractor
Water tank
Sink system
Waste disposal bags
Gloves, Bleach ,methanol
Sputum cups
Pasteur pipettes (spare)
Package material for transport of samples

Forecasting for Commodities

GeneXpert cartridge
Testing of 10% of 180 screened presumptive patients/day requires 18 tests/samples per day
18 tests*5 days*45 wks will require **4,050 Xpert Cartridges.**
40% buffer stock is 1,620
Total cartridge forecasting is 5,670 (114 boxes)
Recording and reporting of Xpert results
According to national guidelines:
GeneXpert machine saves <u>all GeneXpert results</u> and patient data
GxAlert to <u>alert results</u> to clinic, remote control, and summarizing data national wide.
National recording and reporting tool to record results manually.
Dispatch form for transport of samples.
Forecasting for Commodities
CAD4TB
Expected number of persons to be screened
Negotiate for price reduction
Sputum cups

Expected number of persons to be screened
Buffer
Forecasting for Commodities
Electricity
Truck engine alternator
Back up generator/? Solar panel integrated unto the truck
Inverters and batteries
Fuel supply logistics
Forecasting for Commodities

Data management needs

Electronic data management system using national R&R tools to be used
Internet connectivity with cloud back up
Data entry clerks with good computer knowledge
Daily summary of data
Refrigeration needs
GeneXpert cartridges
? Unprocessed samples

Operations:

Logistics at the screening venue (outside the truck)

Canopies/Portable tent
Registration area
Automatic tally dispenser
Result dispatch/post-test counselling area
Chairs

Security

Civil defence corps x 2
Police (if need be)

engagement

Flat screen TV with DSTV connectivity
Lighting arrangement especially at night
Blood pressure measurement and counselling ?
? Take-aways
Drugs (multi-vitamins, etc)
snacks
IEC materials

Human Resources … 1

Screening Coordinator/Team leader x 1
TOR to be developed
Truck driver x 1
Drives and maintains the truck
Provides general support to the support staff as directed
~~Data entry clerks x 2~~
1st registration point

Radiographers x 2
Prepares the client for screening
Operates and maintain the portable x-ray
Operates the CAD4TB software
Ensure general safety procedures in the x-ray room
Lab scientists x 2
Receives and records samples
Runs GeneXpert tests.
Prints out the GeneXpert results
Records GeneXpert results in the register
Hands over the GeneXpert results to the counsellor for dispatch to clients
Takes care of the waste management.
Handle daily, weekly and monthly maintenance of the equipment.
Prepare samples to dispatch for culture/LPA if need be
Human Resources … 3
Support staff
Crowd control officer?
Ensures maintenance of order and provides logistics for seating arrangements etc
Notifies the security agents in case of imminent breach of security
Health educator
Explains the process at the beginning of screening and at intervals
Escort officer?
Escorts presumptive TB cases to obtain sputum sample and submits same to the lab scientist
Counsellor
Gives out GeneXpert results to participants and counsels them accordingly
Links up TB patients to the DOTS officer/TBLS
Human Resources … 4
Support staff
LGTBLs
Direct link between the TB programme and the screening centre
DOTS Officer
Operates a mobile DOTS clinic and put DS-TB patients on treatment
Security
Civil Defence corps members
Police if needed

Linkage to treatment and care

DS-TB
DOTS provider to operate mobile DOTS centers
Register patient in the HF register on site
Commence treatment on site
Make plans for follow up and monitoring of patients
DR-TB
Report of the GxAlert
Notify the STBLCP (STBLCO and DR-TB FP)
Commence treatment as per national guideline

Forecasting for Commodities & Space for mobile Xpert

Peter Nwadike
KNCV/CTB
Mobile Xpert lab in Ghana
How to handle all these samples
.
Testing capacity
.
Testing capacity
.
Sample/Testing Forecasting
.
Supplies needed
.
Laboratory site
Recording and reporting / Connectivity
.
Recording and reporting / Connectivity
.
Electricity capacity
Water supply
Staffing
.
Staff Roles/Relationship
.
Estimated number of tests per technician , WHO guidelines
.
.

Quality Sputum Collection

Priority to obtain quality sputum
Up to 30% of PLHIV and up to 5% of CXR abnormal cannot generate a symptom after positive screen for TB b/c they don't have productive cough.
They would have to be induced – but mobile truck lacks the capacity
Priority to improve the quality of sputum production among PLHIV
The Lung Flute® is an FDA cleared partly re-usable simple horn-shaped plastic device with a vibrating reed that costs under $20.
This CE class I (minimal risk) device produces sound waves using a thin reed and loosens mucus when an individual blows 20 times into the horn.

A hard plastic tube generates a sound with a frequency of 18–22 Hz with an output of 110–115 dB using a pressure of 2.5 cm H2O.
A five minute preparatory breathing exercise has shown effectiveness in specimen production in 88% of adults unable to expectorate independently (Fujita 2009).
New papers in 2014 & 2015
African prev survey sensitivity data women > HIV

TB Screening PLHIV

Ellen M.H. Mitchell
CXR doesn't work so well in PLHIV
CAD4TB doesn't work well with PLHIV
WHO 4 symptom screen
Variation in TB screening among PLHIV.
High sensitivity screen:

1. Any cough,
2. Any fever,
3. Any weight-loss,
4. Any night sweats

Problems with who algorithm in

1. PLHIV pregnant
2. Children
3. PLHIV >500 CD4 c/ ART e TPI

major studies cast doubt on the value of the WHO 4-symptom screen for PLHIV on ART [1-4].

- **Shah S, Demissie M, Lambert L, Ahmed J, Leulseged S, Kebede T, et al.** Intensified Tuberculosis Case Finding Among HIV-Infected Persons From a Voluntary Counseling and Testing Center in Addis Ababa, Ethiopia. Jaids-Journal Acquir Immune Defic Syndr [Internet]. 2009;50(5):537–45. 2.
- **LaCourse SM, Cranmer LM, Matemo D, Kinuthia J, Richardson BA, John-Stewart G, et al.** Tuberculosis case finding in HIV-infected pregnant women in Kenya reveals poor performance of symptom screening and rapid diagnostic tests. JAIDS J Acquir Immune Defic Syndr [Internet]. 2015;71(2):1.
- **Ahmad Khan F, Verkuijl S, Parrish A, Chikwava F, Ntumy R, El-Sadr W, et al.** Performance of symptom-based tuberculosis screening among people living with HIV: not as great as hoped. Aids [Internet]. 2014;28(10):1463–72.
- **Rangaka MX, Wilkinson RJ, Glynn JR, Boulle A, van Cutsem G, Goliath R, et al**. Effect of antiretroviral therapy on the diagnostic accuracy of symptom screening for intensified tuberculosis case finding in a South African HIV clinic. Clin Infect Dis. 2012/09/08 ed. 2012;55(12):1698–706.

CXR screening in PLHIV requires add'l skill—

BUT since we'll be at Health centers, we'll leverage available expertise in site
Xpert also doesn't work as well in PLHIV
Sensitivity of only 77% (Steingart et al 2015)
Apply different screens to different types of PLHIV?
'Where there is no CD4 count machine'
Most of the TB screening and IPT provision needs to occur in "last mile settings" where :

1. patients may not have their CD4 count yet
2. may not have access to real time viral load and CD4 testing.

3. TB screening should occur with more frequency that CD4 re-testing.

Variable TB screens are needed that can take into account uncertainty.

Can TB symptoms screens be improved so they can work better in ACF?

Maybe. There are some ideas to link symptoms screening with risk factor analysis. However this work is all exploratory at this stage.

Sample TB Screening Scoring

Use of Risk Factors to compliment symptoms

Malnutrition (RR 3.2)

BMI – need a scale and height measure

Mid-Upper Arm Circumference

MUAC

Mid-Upper Arm Circumference – relationship to HIV and TB?

But:

In Tanzania, HIV+ pregnant women with a MUAC< 22mm had a HR of **2.4** (95%CI1.2-4.6) for incident TB (Venkatesh, 2005)

Among TB patients in Guinea Bissau Adult MUAC of ≤ 21mm had sensitivity 54%, specificity 76% for TB Mortality (Gustafson et al 2007)

It is used now as a warning sign for non-response to TARVs (Maman et al,2012)

ANEMIA as predictor of TB in PLHIV

ANEMIA!

Visual inspection for Oral candida?

We know it is a good measure of immune suppression

Urine LAM is a good test for immunesuppressed PLHIV

Determine TB-LAM diagnostic assay???

Perhaps, but not in the truck - yet. Better to start off in hospitals

Detects Pulmonary and extra-pulmonary TB

Takes a drop of urine

Results in 25 minutes

Nasarawa state

Principles of Active Case Finding – Strategic Choices and Prioritization Situational Assessment

Project Areas

1. Karu LGA
2. Nasarawa LGA
3. Keffi LGA
4. Nasarawa-Eggon LGA
5. Lafia LGA

2015 Estimated adult population (≥ 15 years) of the 5 LGAs by age groups

2015 Estimated adult (≥ 15 years) urban slum population of the 5 LGAs					
	Adult (≥ 15 years) population	Urban adult population	Urban adult slum population	Percentage	Remarks
Karu	153,610	92,166.18	36,866	34%	60% of adult population live in the urban areas out of which 40% live in slums
Keffi	65,748	39,448.64	15,779	15%	60% of adult population live in the urban areas out of which 40% live in slums
Lafia	234,377	117,188.68	29,297	27%	50% of adult population live in the urban areas out of which 25% live in slums
Nasarawa	133,002	66,500.77	16,625	16%	50% of adult population live in the urban areas out of which 25% live in slums
Nasarawa Eggon	105,427	42,170.91	8,434	8%	40% of adult population live in the urban areas out of which 20% live in slums
Total of 5 LGAs	**692,164**	**357,475**	**107,002**	**100%**	

Main Risk Groups in Nasarawa State

1. Slum Dwellers:
   - Nyanya
   - One Man Village
   - Ado village
   - Masaka
   - New Karu
   - Uke
2. Transport workers, Automobile and Construction workers
3. Large Scale farms
4. Sharna Farms
5. Abdullahi Adamu's Farm
6. Mega worship centres

7. Goshen
8. Redeemed
9. Mosque by Masaka Market
10. Emirs palace mosques
11. IDPs
12. Alcohol Abusers
13. Zimbabwe
14. Shopping center, One Man village

Risk Groups	LGAs	Estimated population	Population Participating	Prev rate (per 100,000 popn)	Estimated TB burden among screened	Diagnosed TB cases (80% sensitivity)	Comments and Assumptions
Urban Slum dwellers that are ≥ 15 years	Karu	36,866	20,645	663	137	110	56% of the popn accepts screening (participation rate of the nat prev survey). Using urban prevalence rate of the TB prevalence survey
	Keffi	15,779	8,836	663	59	47	
	Lafia	29,297	16,406	663	109	87	
	Nasarawa	16,625	9,310	663	62	49	
	Nasarawa Eggon	8,434	4,723	663	31	25	
	Sub-Total	107,002	59,921	3,315	397	318	
IDPs	Karu	2500	1,400	663	9	7	
	Sub-Total	2,500	1,400	663	9	7	
Mega worship centres	Karu	15,000	3,000	323	10	8	20% of the popn accepts screening
	Keffi	4,000	800	323	3	2	
	Sub-Total	19,000	3,800	646	12	10	
Risk Groups	LGAs	Estimated population	Population Participating	Prev rate (per 100,000 popn)	Estimated TB burden among screened	Diagnosed TB cases (80% sensitivity)	Comments and Assumptions
Transport/Automile and ocnstruction workers	Karu	3000	1,800	663	12	10	56% of the popn accepts screening (partiicpation rate of the nat prev survey). Using urban prevalence rate of the TB prevalence survey
	Keffi	1500	900	663	6	5	
	Lafia	2000	1,200	663	8	6	
	Sub-Total	6,500	3,900	1,989	26	21	
Large farms	Karu	2,000	1,200	663	8	6	
	Keffi	1,500	900	663	6	5	
	Sub-Total	3,500	2,100	1,326	14	11	
Alcohol dependent	Karu	2,000	1,200	663	8	6	
	Lafia	800	480	663	3	3	
	Nasarawa Eggon	1,000	600	663	4	3	

	Sub-Total	3,800	2,280	1,989	15	12	
TOTAL		142,302	73,401	9,928	474	379	

2015 Estimated adult (≥ 15 years) urban slum population of the 5 LGAs

Population of main Risk Groups in Nasarawa State

Selection of Risk groups for prioritization

Current distribution of TB burden in the LGAs

Current distribution of TB burden in this setting (for the different sub populations)

Main reasons for Gap in TB case detection

Patient related factors:

- Limited awareness about TB and TB services points
- Lack of money
- Denial
- Fear of stigmatization

Health Care factor:

- Limited knowledge about TB
- Inadequate number
- Poor attitude to work
- Parallel charges by health workers

Main reasons for Gap in TB case detection

- Health system factors:
- Out of commodities
- Limited to access to service
- Frequent industrial actions

Programme related factors:

- Poor documentation or poor data quality
- Weak monitoring

Age-Sex distribution of Bact. confirmed TB cases in karu LGA

Estimated versus Notified cases

Male

Female

Age-Sex distribution of Bact. confirmed TB cases in keffi LGA

Estimated versus Notified cases

Male

Female

Age-Sex distribution of Bact. confirmed TB cases in Laffia LGA

Estimated versus Notified cases

Male

Female

Age-Sex distribution of Bact. confirmed TB cases in Nasarawa LGA

Estimated versus Notified cases

Male

Female

Age-Sex distribution of Bact. confirmed TB cases in Nas. Eggon LGA

Estimated versus Notified cases

Male

Female

Stakeholder Analysis

Community Leaders

1. Etso Karu
2. Chief Imam of Karu
3. Catholic Dioceses
4. Winners, Redeemed, ECWA, COCIN
5. NURTW Chairperson
6. Youth Groups
7. CBOs
8. Landlords and Neighborhood Association
9. Stakeholder Analysis
10. Security organizations
11. Nigeria police
12. NSCD
13. NPS
14. Road Safety
15. Nigeria Vigillante
16. Stakeholder Analysis
17. Health Service Providers:
18. Patent Medicine Vendors (PMVs)
19. Ward Development Health Committees (WDCs)
20. HOD of health in the LGA
21. Facility heads of existing TB service delivery points
22. Local MMA, GMDs, AGPMPN
23. Local MLSCN
24. Local NMAN
25. TBLS
26. DOTS Providers and AFB Microscopist and GeneXperts.
27. Stakeholder Analysis
28. Government:
29. State Ministry of Health
30. Local Government authority
31. HOD Health
32. PHCDA
33. Govt. Structures in charge of market and park
34. Health councilors
35. Chairmen of Local government Development Areas
36. Stakeholder Analysis

Media:

1. Community Radios
2. Brekete family radio

Other high level media engagements
Potential Sites for Screening
How and Where?
Pre-implementation assessment

NASARAWA LINKAGE OF DSTB, DRTB AND HIV CLIENTS TO SERVICE CENTERS

Aboki, D.M.
(STBLCO)
LINKAGE TO CARE
DS-TB
DR-TB
HTC
For DS-TB Patients
At the screening Centre:
DOTS provider supported by TBLS
Facility TB register
Drugs
Weighing scale
Treatment cards
DOTs provider care contd.
-Puts the TB patient on treatment
Registers patient
Fills the treatment card
Follow up with investigation and monitoring at DOTs centre
DR- TB patient
Following diagnosis of RIF+
Gx Alert>STBLCO, DRTB FP,& National TB Program
Daily notify>STBLCO, DRTB FP,& TBLS
STBLCP work up patient for treatment
STBLCP put patient on treatment(Facility/ Community)
Follow up to ensure patient is put on treatment

CHALLENGES TO TX INITIATION/ADHERENCE

Treatment Initiation:

- Denial/Refusal to take treatment
- Counseling at site

Follow up with influencers(traditional rulers)
Use ex-TB patients as educators

Unclaimed results

TBLS and CBOs to trace patient
? Incentives

Drug stock out

- Very good logistic management information system
Challenges Initiation/Adherence cont.
Adherence:

- Follow up and monitoring at a designated AFB and DOTS center
- Prioritize DOTS centers for routine supervision
- Continuous health education

HTC AND ART

For presumptive TB clients – No HTC on the trucks

For TB cases- HTC at DOTS site

Is the TB treatment infrastructure sufficient?

NASARAWA STATE TB/LEPROSY/BURULI ULCER CONTROL PROGRAMME

UPDATED DOTS AND MICROSCOPY CENTRES

S/NO	LOCAL GOVT AREA	DOTS CENTRE	MICROSCOPY CENTRE
1	Lafia	- DASH Lafia<br>- Model Clinic Tudun Kauri<br>- PHC Centre M/Akunza<br>- PHC Centre Kwandere<br>- PHC Centre B.A.D<br>- Nig Prison Service Lafia<br>- Model Health Centre Shabu<br>- PHC Doma Road Lafia<br>- Taimako Clinic Assakio<br>- Ekoson Maternity Clinic Bukan Koto<br>- Voice of Islam Hospital Lafia	- DASH Lafia<br>- PHC Clinic Kwandere<br>- Model Clinic Kwandere<br>- Model Clinic Shabu
2	Akwanga	- Gen Hosp Akwanga<br>- PHC Centre Andaha<br>- PHC Clinic Ungwan Zaria<br>- PHC Ungwan Zaria<br>- PHC Clinic Bayan Dutse<br>- OLA Hospital Akwanga<br>- PHC Clinic Moroa<br>- Royal Hospital Akwanga<br>- Mochu Memorial Clinic Moroa	- Gen Hosp Akwanga<br>- PHC Centre Andaha<br>- OLA Hospital Akwanga
3	Keffi	- Federal Med Centre Keffi<br>- PHC Centre Angwan Waje Keffi<br>- Innovative Biotech Keffi<br>- General Hospital Keffi<br>- Army Barracks Keffi	- Federal Med Centre Keffi
4	Nasarawa	- Gen Hosp Nasarawa<br>- PHC Emir's Palace Nasarawa<br>- PHC Loko<br>- PHC Ara 1<br>- PHC Centre M/Udege	- Gen Hosp Nasarawa<br>- PHC Centre Loko<br>- PHC Emir's Palace Nasarawa<br>- PHC Centre Udeni Kasa

		- PHC Udeni Gida<br>- PHC Udeni Kasa<br>- PHC Laminga<br>- Nasarawa Med Centre<br>- Alumbugu Memorial Clinic Kurudu<br>- Rizizei Clinic Ajaga<br>- Alpha Hospital Nasarawa<br>- Nigeria Prison Service Nasarawa	- PHC Centre Udeni Gida<br>- PHC Centre Laminga

S/NO	LOCAL GOVT AREA	DOTS CENTRE	MICROSCOPY CENTRE
5	Karu	- Med Centre M/Gurku<br>- Gen Hosp Panda<br>- Gen Hosp Uke<br>- PHC Centre Masaka<br>- PHC Gunduma<br>- PHC Gitata<br>- PHC Takalafiya	- Med Centre M/Gurku<br>- Gen Hosp Panda<br>- Gen Hosp Uke<br>- PHC Gunduma<br>- PHC Gitata
6	Awe	- Gen Hosp Awe<br>- PHC Centre Azara<br>- PHC Centre Tunga	- Gen Hosp Awe<br>- PHC Centre Azara<br>- PHC Tunga
7	Keana	- Gen Hosp Keana<br>- Model PHC Centre Kadarko<br>- PHC Centre Uluji<br>- PHC Centre Kwarra<br>- PHC Giza	- Gen Hosp Keana<br>- Model PHC Centre Kadarko
8	Wamba	- Gen Hosp Wamba<br>- PHC Centre Arum Sarki<br>- PHC Centre Nakere<br>- SDA Hospital Arum Tumara<br>- PHC Centre Kwara<br>- PHC Centre Marhai<br>- PHC Centre Kwabe<br>- PHC Centre M/Gongo<br>- ERCC Dispensary Konva	- Gen Hosp Wamba<br>- PHC Centre Arum Sarki<br>- SDA Hospital Arum Tumara<br>- PHC Centre Kwara
9	Toto	- Gen Hosp Toto<br>- PHC Centre Gadabuke<br>- PHC Centre Karmo<br>- PHC Centre Ugya<br>- Cottage Hospital Umaisha	- Gen Hosp Toto<br>- PHC Centre Gadabuke<br>- Cottage Hospital Umaisha
10	Obi	- Gen Hosp Obi<br>- PHC Centre Adudu<br>- BHC Duduguru	- Gen Hosp Obi<br>- PHC Centre Agyaragu

		- PHC Centre Agwatashi<br>- PHC Adudu<br>- PHC Centre Agyaragu<br>- PHC Centre Gidan Ausa<br>- PHC Centre Dadere	
11	Doma	- Gen Hosp Doma<br>- PHC Centre Burum-Burum<br>- PHC Agbashi<br>- PHC Rukubi<br>- PHC Igbabo<br>- PHC Bosco Road Doma<br>- PHC Doma Town	- Gen Hosp Doma<br>- PHC Centre Agbashi<br>- PHC Centre Rukubi

S/NO	LOCAL GOVT AREA	DOTS CENTRE	MICROSCOPY CENTRE
12	Nasarawa Eggon	- ERCC Hospital Alushi<br>- Gen Hosp Nas/Eggon<br>- PHC Centre Mada Station<br>- PHC Centre Wowyen<br>- PHC Centre Bakyano	- ERCC Hospital Alushi<br>- PHC Centre Mada Station<br>- Gen Hosp Nas/Eggon<br>- PHC Centre Wowyen<br>- PHC Centre Bakyano
13	Kokona	- Gen Hosp Garaku<br>- PHC Centre Agwada<br>- PHC Centre Bassa	- Gen Hosp Garaku<br>- PHC Centre Agwada<br>- PHC Centre Bassa

Key populations and Potential Screening sites in Ogun State

Dr F.O.Soyinka

Urban slums

LGA	Name	Number of inhabitants	Number of DOTS sites within 1 km
Ado Odo/Ota	Ketere Sango	7,800	2
Ado Odo/Ota	Araromi 1&2	100,000	3

Ado Odo/Ota	Ajegunle	20,000	3
Yewa South	Ibese	??	0

LGA	Name	Number of inhabitants	Number of DOTS sites within 1 km
IFO	Igboko, Leyun, Midan Olowe1,2,3. Muta	Over 2,000	0
IFO	Akute, Abule Ojo, Onibudo Ganwin, Abule Ofin, Dele Ogundele, Adiyan, Iseri Olofin, Grail Land	Over 2,500	0
IFO	Isuren Obagimu, Oseni,Karonwi	Over 1000	0
IFO	Abule Sule Risere	Over 500	06

Urban slums

Football stadiums

Name	LGA	Size of stadium	Average attendance	Match frequency	Link to DOTS centers?	Safety/security
Sango Stadium	Ado odo ota	105 by 68 meters	1000	Once in a month aveeragely	yes	yes

Sagamu Stadium	Sagamu					

High Risk Occupations

Type of work	Location	Size of workforce	Number of DOTs sites within 1 km
Cement Factory	1Dangote, Ibese. (YS)<br>2 Lafarge Sagamu(Sag)<br>3.Lafarge Ewekoro 1&2	1)>2000 +Casual Workers (CW)<br>2) 194 + CW<br>3) 376+ CW	0
Mechanic Villages	Sagamu<br>Ota ??<br>Ifo??<br>Ilaro??		
Motor Garages	Sabo, Ogijo (Sagamu)<br>Mowe, Ibafo (Obafemi Owode)<br>Ifo, Sango (Ado Odo/Ota)		

Type of work	Name of company	Size of workforce	Number of DOTs sites within 1 km

Quarry Miners			
Wine Distillers			

Transport Centers- bus depots

LGA	Name	Approx. size of population daily
Obafemi Owode	Mowe, Ibafo	?
Sagamu	Sabo, Ogijo	?
Yewa South	Ilaro	?
Ifo	Ifo Garage	250
	Ifo Market	3,000
Ado Odo/Ota	Sango garage	20,000
	Bridge garage	15,000
	Ijoko garage ntabo	10,000
	Atan garage	7000

Centers for Vulnerable Youth –remand ctrs

Name	LGA	# residents	staff

Ijamido	Ado odo ota	30	8

HIV clinics

LGA	NAME	PLHIV Caseload	% of caseload on ART	Patient volume (daily)
Ado Odo/Ota	State Hosp Ota	1500	100%	10
	Ota Health Centre	100	100%	5
Yewa South	State Hospital Ilaro			
Ifo	General Hosp Ifo			
Ifo	PHC Olose			
Obafemi Owode	General Hospital Owode			
Sagamu	OOUTH Sagamu			

HIV prevalence **Ogun state**

Population : 4949993
HIV Prevalence: 2.9%
TB CNR: 50.2/100,000

Private and mission health facilities

LGA	NAME	Out-patient volume (daily)	DOTS clinic	On site CXR?
Yewa South	St Mary Catholic Clinic, 2) Hossanah Specialist	?	Yes	?
Obafemi Owode	Redemption Camp Health Centre	?	Yes	?
Ado Odo/Ota	1) Ace Medicare, 2)		Yes	Yes(1)
Ado odo ota	Catholic Hospital Ado ado odo	30	No	No
Ado odo ota	Convenant University hospital	25	No	No
Ado odo ota	Mulib Hospital	20	Yes	No
Ado odo ota	Hetta Hospital	35	No	No
Sagamu	Redemption Hosp, Owokoniran memorial Hosp		Yes	No

Prisons
Diabetes clinics

Ogun Team Priority LGAs (2015 est. population)

Ado-Odo Ota - 698,827
Yewa South – 223,119
Obafemi Owode – 311,572
Ifo – 714,637
Sagamu – 339,160

Prioritized Risk groups

1. **Slum inhabitants** (18 locations identified)
2. **Contacts of DSTB & DRTB patients**
3. **Silica Miners**
- Cement factories (2 locations identified)
- Quarries
1. **Out patients**
- Medical outreach
- Diabetics clinics
- Children clinics

2. **Men**

- Mechanic Villages (2 locations identified)
- Motor parks

3. **Nomads**
4. Sabo communities in Sagamu
5. Prisons (2/5 locations identified)

LGA	Name	Inmate Population	# of staff	TB screening?	TB caseload 2015
Yewa South	NPS Ilaro			Yes	0
Sagamu	NPS Sagamu	256	43	Yes	4
Obafemi Owode	NPS Oba			Yes	0

Diabetes Clinics

LGA	Name	Patient volume (daily)

Sagamu	OOUTH Diabetic Clinic	
Ado Odo/Ota	State Hosp Ota	

Population of identified Risk groups- Ogun

LGA	Name	Number of inhabitants	Number of DOTS sites within 1 km
Ifo	Akute, Ajuwon		
Obafemi Owode	(1) Mowe, (2) Ibafo (3) Ofada	(1) 68,871, (2) 13,931 (3) 13,855	1, 1,1
Sagamu	Sabo Sagamu	?	1
Sagamu	Ogijo Community	?	1

Gaps in Case detection

Reasons for Gaps in Case Detection in Ogun

Community Level

1) Low awareness of TB symptoms and location of services
2) 60% gap in AFB microscopy labs (1/50,000)
3) 50% gap in Xpert labs (1/500,000)
4) Uneven/Skewed distribution of diagnostic services
5) Poor health seeking behavior (PMVs, Traditionalist, religious leaders)
6) Long distance to available services (poor access)
7) Stigma

Facility Level

1) Low awareness of HCWs on TB management, available services (none DOTS/DOTS/diagnostic)
2) Low index of suspicion for pulmonary TB and EPTB
3) Availability of HCWs & diagnostic services
4) Delay in collection of samples
5) Delay in release/receipt of lab results
6) Undocumented lab results (initial lost to follow up)
7) Unavailable/interrupted supply of drugs/consumables

8) Weak hospital-DOTS linkage
9) Weak sputum referral linkage
10) QA (Quality Assurance) procedure in lab diagnosis (High false negative)

Main Partners for Key populations in Ogun

1. SMoH,
2. Community Leaders,
3. CBOs,
4. TBLS,
5. LGA Authorities,
6. GHWs (in adjoining HF),
7. PMVs
8. Private partners (DFB, OASIS Foundation),
9. SASCAP/LACA,
10. Media,
11. Industries,
12. Road transport workers,
13. Association of Automobile technicians

Algorithm

Linkage to care is guided by the Algorithm

1. Clients requiring care;
2. Abnormal Xray findings not suggestive of TB e.g FB
3. Abnormal Xray suggestive of TB (≥ 60)
4. Abnormal Xray plus Xpert positive
5. Xpert positive normal Xray (symptomatic)

ABNORMAL FINDINGS (NOT TB)

Mapping of available health service within selected sites e.g specialist centres, secondary Health facilities & private facilities (strike)
Advocacy to mapped sites.
Referral

MATCHING THE SCREENING SITE TO TREATMENT SITE

1. Identify facility to manage patients using LGA directory
2. Engaging TBLS within selected LGA
3. Identify facility with low TB burden to participate.
4. Develop DOTS directory for each of the selected LGAs.
5. Introduce participant initiated treatment initiation site
   Option 1 – facility serving mobile ACF.
   Option 2 – Selection from directory.

M&E Framework

The ACF pilot in Ogun and Nasarawa will be conducted in an operational research framework to ensure timely information on the cost-effectiveness, yield, feasibility, and acceptability. The consultant will ensure an adequate M&E envelope is in place to provide data for decision making on scale up.

Monitoring, evaluation and re-programming

Defined indicators to monitor:

- ☐ Yield, NNS, confirmed vs. non-confirmed cases
- ☐ Contribution to case notification (immediate and at 1 yr post screening)
- ☐ Cost and cost per case
- ☐ Minimum recording and reporting requirements
- ☐ Number screened
- ☐ Number screened positive on CXR or symptoms
- ☐ Number tested
- ☐ Number pulmonary B+ TB patients detected (by type)
- ☐ Number of pulmonary B+ TB patients who return to pick up their GXP results
- ☐ Number of Pulmonary B+TB patients with pre-treatment Loss to follow up (untraceable)
- ☐ Number Bac+ TB patients who start treatment
- ☐ Number of presumptive clients with Abnormal CXR and NEG GXP who enroll our " follow up program" (i.e. SMS messaging, and 12 mos re-testing) to capture incipient TB.
- ☐ Number Needed to Screen (NNS) to find 1 true case – (same-day dx)
- ☐ Number Needed to Screen (NNS) to find 1 True case – (same-day dx + later cases)

GXConnect wireless dual sim router will be connected to all the GeneXpert computers to upload test results to the GXAlert server. We are in good communication with the organisation that deploy this device in Nigeria (Abts Associate).

Evidence-based active case finding (ACF) to identify the missing cases in Nigeria is the top priority of the Nigerian TB program and is aligned with the goals of Challenge of TB and GFATM. ACF can be very

useful but if poorly targeted or executed, it can be a costly exercise. Active case finding using mobile Chest-x-ray/Xpert vehicles will be implemented in Ogun and Nasarawa states in APA3. The mission involved a broad stakeholder consultation to define the target populations, screening algorithm(s), treatment initiation steps, and follow up procedures for the cohort with CXR abnormalities. The consultant will provide the team with evidence-based options so that the pros and cons of different operational choices are clear. The 7-step process for this planning workshop was derived from the WHO Operational Guidance on Screening (June, 2015) which is an inclusive, sequential approach to reaching decisions on what, where, when and how to do effective ACF.

WORKSHOP RECOMMENDATIONS

The next steps and recommendations form the workshop are described in this table with suggested responsible persons indicated.

Recommendations	Actions	Responsible person
1. Timely purchase of the CXR/Xpert mobile screening trucks	• The tender for the trucks must be published immediately by het NIC	Jan Willem Dogger
2. Obtain consensus and approvals from all stakeholders for the chosen approach –	• USAID sign-off on expected yield and cost per case<br>• KNCV Technical Unit needs to approve the use of CAD4TB without the calculation of diagnostic accuracy.<br>• Talk to WHO ACF team	Rupert/ Ellen Nkem/Gidado/Michael Kimerling
3. Engage OLD DELFT the CAD4TB copyright holder	• to see if we can interest them in testing a potential new advantage of their technology, in exchange for subsidizing the study – looking at the predictive value of the technology.<br>• negotiate early to obtain lowest unit price for CAD4TB. We do not want to pay $1.25. We want the prevalence survey price ($1.0) or lower. This is critical for cost effectiveness and sustainability .	Jan Willem Dogger/ Ellen Mitchell
4. Engage FIND and CEPHEID to obtain early access to Ultra	• Contact FIND to determine if this exercise would qualify for the use of the new Ultra cartridges.<br>• This is critical for cost effectiveness and sustainability.	Jerod Scholten/ Petra de Haas
5. Refine operational and management plans	• Seek and obtain sign-off on the operational plans<br>• Develop SOPS and training manuals order commodities, set timelines,<br>• clarify roles and responsibilities	Rupert Enugu, Peter, Johan,

6. Build staff capacity to execute with fidelity and excellence	• Develop training curriculum for CXR and lab staff<br>• Develop job descriptions, recruit,	Rupert Enugu
7. Engage the target populations (high risk sites and pops)	• Map the treatment initiation pathways for all the screening areas (e.g. mechanics' villages, urban slums)	Rupert/ nkem
8. Refine the M&E system	• Determine how to map the eligible population<br>• Pre-test M&E tools<br>• Develop digital data entry system on tablets –<br>• Figure out integration with GeneXAlert.<br>• Figure out how the cohort of clients without current TB but with CXR abnormalities will be followed (via SMS?) and how their subsequent TB testing and DX data will be collected<br>• Determine the tools for costing information will be collected	Hadi/ Chidubem, Jumoke

APPENDICES

MOBILE CXR SCREENING DAILY SUMMARY

Date: ___________ Location:______________ Population: __________

Today's screening target: ___________ Today's case-finding target:

Total CXR Screens Achieved:__________ Total symptom screens achieved: ____________

Total Presumptive clients Found:_____ Total clients tested: _____

Total clients with valid results: _____ Total clients post-test counselled: ______

Total referred for 6 month follow-up for ABNORMAL CXR: _________

CXR SCREENED

	20-29yr	30-39yr	40-49yr	50-59yr	60-69yr	≥70yr
HIGH RISK MALES						
HIGH RISK FEMALES						

GENERAL MALES						
GENERAL FEMALES						

(LOW RISK CXR SCREENED

	0-4yr	5-9 yr	10-14 yr	15-19 yr
SYMPTOMATIC FOR TB				
OTHER				

PRESUMPTIVE CLIENTS IDENTIFIED BY CXR

	20-29yr	30-39yr	40-49yr	50-59yr	60-69yr	≥70yr	Total
HIGH RISK MALES							
HIGH RISK FEMALES							
GENERAL MALES							
GENERAL FEMALES							

	0-4	5-9	10-14	15-19	Total
SYMPTOMATIC FOR TB					
OTHER					

PRESUMPTIVE CLIENTS WHO PRODUCED A SAMPLE FOR TESTING WITH XPERT

	20-29yr	30-39yr	40-49yr	50-59yr	60-69yr	≥70yr	Total
HIGH RISK MALES							
HIGH RISK FEMALES							

GENERAL MALES							
GENERAL FEMALES							

CLIENTS POST-COUNSELLED WITH VALID XPERT RESULTS

	20-29yr	30-39yr	40-49yr	50-59yr	60-69yr	≥70yr	TOTAL
HIGH RISK MALES							
HIGH RISK FEMALES							
GENERAL MALES							
GENERAL FEMALES							

XPERT POSITIVE TB CASES REFERRED FOR TB TREATMENT

	20-29yr	30-39yr	40-49yr	50-59yr	60-69yr	≥70yr	total
HIGH RISK MALES							
HIGH RISK FEMALES							
GENERAL MALES							
GENERAL FEMALES							

XPERT NEGATIVE PRESUMPTIVE CLIENTS REFFERED FOR FURTHER CLINICAL INVESTIGATIONS

	20-29yr	30-39yr	40-49yr	50-59yr	60-69yr	≥70yr	TOTAL
HIGH RISK MALES							
HIGH RISK FEMALES							

GENERAL MALES							
GENERAL FEMALES							

XPERT NEGATIVE PRESUMPTIVE CLIENTS ENROLLED IN FOLLOW-UP ALERT PROGRAM FOR MANAGEMENT OF ABNORMAL X-RAY

	20-29yr	30-39yr	40-49yr	50-59yr	60-69yr	≥70yr	TOTAL
HIGH RISK MALES							
HIGH RISK FEMALES							
GENERAL MALES							
GENERAL FEMALES							

DRAFT QUESTIONNAIRE FOR PERSONS WITH GXP+ TB RESULTS

Check one: Date: ___ / ___ / ___

Location code: ______

INFORMATION TO READ TO RESPONDENT:

We wish to learn about your knowledge, attitudes and practices regarding tuberculosis (TB). We hope to understand your needs and the best way to bring information to you, as well as barriers to seeking medical care. The information you provide will be used to improve TB control.

Your answers will not be released to anyone and will remain anonymous. Your name will not be written on the questionnaire or be kept in any other records.

Your participation is voluntary and you may choose to stop the interview at any time.

Thank you for your assistance.

SECTION 1: GENERAL AND DEMOGRAPHIC QUESTIONS

1. How old are you?

1	Under 30 years	

2	31 – 40 years	
3	41 – 50 years	
4	Over 50 years	

2. What is your gender?

1	Male	
2	Female	

3. What is the highest level of education you have completed?

1	No school	
2	Primary school	
3	Junior secondary school	
4	Senior secondary school	
5	Undergraduate education	
6	Post graduate education	
7	Religious schools	
8	Literacy classes	

4. Do you currently have paid employment?

1	Yes	
2	No	

5. What is your occupation?

____

6. How far do you live from the nearest health clinic or hospital?

1	0 – 10 km	
2	11 – 20 km	
3	21 – 30 km	
4	More than 30 km	

7. Marital status

Single	

Married	
Separated	
Divorced	
Widowed	
Others (Please specify)	

SECTION 2: HEALTH-SEEKING BEHAVIOUR

8. Where do you usually go if you are sick, or to treat a general health problem? (Check all that are mentioned.)

1	Private clinic	
2	Government clinic or hospital	
3	Traditional or homeopathic healers	
4	Clinic run by a Non-Governmental Organization (NGO)	
5	Church	
6	I do nothing	
7	Others (Please specify)	

9. How often do you generally seek health care at a clinic or hospital? (Check one.)

1	Twice a year	
2	Once a year	
3	Less than once per year but at least twice in the past 5 years	
4	Once in the past 5 years	
5	Never in the past 5 years	
6	Others (Please specify)	

SECTION 3: TB KNOWLEDGE AND AWARENESS

10. Have you ever heard about tuberculosis?

1	Yes	
2	No	

11. Where did you first learn about tuberculosis or ‘TB’? (Check all that are mentioned.)

1	Newspaper and magazines	
2	Radio	
3	TV	
4	Billboards	
5	Brochures, posters and other printed materials	
6	Health workers	
7	Family, friends, neighbors and colleagues	
8	Religious leaders	
9	Teachers	
10	Others (please explain)	

12. In your opinion, how serious a disease is TB? (Check one.)

1	Very serious	
2	Somewhat serious	
3	Not very serious	

13. How serious a problem do you think TB is in your country/region? (Check one.)

1	Very serious	
2	Somewhat serious	
3	Not very serious	

14. What are the signs and symptoms of TB? (Please check all that are mentioned.)

1	Rash	
2	Cough	
3	Cough lasting for more than 2 weeks	
4	Coughing up blood	
5	Severe headache	
6	Nausea	
7	Weight loss	
8	Fever	
9	Fever without clear cause lasting for more than seven (7) days	
10	Chest pain	
11	Shortness of breath	
12	On going fatigue	
13	Weight loss	
14	Night sweat	
15	I don’t know	
16	Others (please specify)	

15. How can a person get TB? (Please check all that are mentioned.)

1	Through handshakes	
2	Through the air when a person with tuberculosis coughs or sneezes	
3	Through sharing of dishes	
4	Through eating from the same plate	
5	Through touching items in public places (door knobs, handles in transportation, etc)	
6	I don’t know	
7	Others (please explain)	

16. How can a person prevent getting TB? (Please check all that are mentioned.)

1	Avoiding handshakes	
2	Covering mouth and nose when coughing or sneezing	
3	Avoid sharing dishes	
4	Washing hands after touching items in public places	
5	Closing windows at home	
6	Through good nutrition	
7	By praying	
8	I don’t know	
9	Others (Please explain)	

17. In your opinion, who can be infected with TB? (Please check all that are mentioned.)

1	Anybody	
2	Only poor people	
3	Only homeless people	
4	Only alcoholics	
5	Only drug users	
6	Only people living with HIV/AIDS	

7	Only people who have been in prison	
8	Others (Please explain)	

18. Can TB be cured?

1	Yes	
2	No	
3	1 don't know	

19. How can someone with TB be cured? (Check all that are mentioned.)

1	Herbal remedies	
2	Home rest without medicines	
3	Praying	
4	Specific drugs given by health centres	
5	Taking complete TB treatment	
6	I don't know	
7	Others	

20. What are the sources of information that you think can most effectively reach people like you with information on TB? (Please choose the three most effective sources.)

1	Newspapers and magazines	
2	Radio	
3	TV	
4	Billboards	
5	Brochures, posters and other printed materials	
6	Health workers	

7	Family, friends, neighbours and colleagues	
8	Religious leaders	
9	Teachers	
10	Others (Please explain)	

21. What worries you the most when you think about TB?

THANK YOU VERY MUCH FOR PARTICIPATING

Energizer exercise – positioning ourselves for A C F planning

This exercise was used to explore our values and feelings with regard to the benefits and costs of active case finding. Participants were asked to read these statements aloud and to position themselves in front of signs reading: strongly agree, agree, disagree, strongly disagree, and not sure/don't know. The objective was to get up and move –but also to appreciate the sometimes difficult choices and strong opinions that can surround TB active case finding. The exercise was successful in getting participants to stretch their minds and legs – and it catalyzed some very interesting discussions about the project.

Statements

1. TB IN NIGERIA IS URBAN
2. TWICE AS MANY NIGERIAN MEN HAVE TB AS WOMEN
3. WOMEN WITH TB ARE MORE LIKELY TO BE DIAGNOSED THAN MEN WITH TB
4. NIGERIAN PRISONS ARE SMALL
5. TB IN PLHIV IS HARD TO DIAGNOSE EVEN WITH X-RAY AND XPERT
6. DIABETES IN COMMON IN NIGERIA BUT MOST PEOPLE DON'T KNOW THAT THEY HAVE IT
7. HEALTH WORKERS ARE RELUCTANT TO TEST THEMSELVES FOR TB DUE TO STIGMA
8. FEW MEN ATTEND HEALTH FACILITIES BECAUSE IT IS FULL OF WOMEN
9. HAVING AN X-RAY CAN BE FRIGHTENING FOR HEALTHY PEOPLE
10. A FREE X-RAY WOULD BE AN INCENTIVE FOR PEOPLE TO PARTICIPATE
11. ACTIVE CASE FINDING MAY MISS 10-20% OF THE TB CASES AND STILL BE EFFECTIVE
12. ACTIVE CASE FINDING REDUCES THE COSTS THAT PEOPLE NEED TO PAY FOR DIAGNOSIS
13. IF MANY PEOPLE WITH TB RECEIVE LIFE SAVING BENEFIT FROM OUR SCREENING INTERVENTIONS, BUT SOME PEOPLE ARE PUT ON TB TREATMENT BY MISTAKE, THE BENEFIT TO THE MANY OUTWEIGHTS THE HARM TO THE FEW.

14. WE HAVE A HIGHER ETHICAL RESPONSIBILITY TO HELP PEOPLE WHEN WE REACH OUT TO THEM IN THE COMMUNITY, THEN WHEN THEY GO THEMSELVES TO A HEALTH FACILITY.

15. PUBLIC HEALTH IS ABOUT ENSURING EQUAL OPPORTUNITY OF ACCESS NOT TARGETTING RESOURCES TOWARDS COMMUNITIES MOST AFFECTED.

16. IF WE TELL PEOPLE WITH ABNORMAL CXT THAT THEY NEED TO RE-TEST IN 6 MONTHS, THEY WILL NOT DO IT.

17. THERE IS NO SUCH THING AS A PERFECT TEST.

18. EVEN IF SCHOOL CHILDREN HAVE THE LOWEST TB RATES OF ANY GROUP, WE SHOULD STILL SCREEN THEM BECAUSE THEY ARE A LARGE GROUP AND POTENTIALLY VULNERABLE.

19. IT IS BETTER TO BE TOLD THAT YOU DO NOT HAVE TB WHEN YOU DO, THEN TO BE PUT ON TREATMENT WHEN YOU DON'T NEED IT.

20. SCREENING PREGNANT WOMEN WITH CHEST X-RAY IS TOO RISKY EVEN IF LEAD ABDOMINAL SHIELDS ARE WORN.

Participants list

ACF workshop Participants

S/N	**Name of participants**	**Designation**	**Organization**
1	Dr Omoniyi Fadare	TB/HIV Advisor	WHO
2	Dr Enang Oyama	Health System Strengthening Advisor	WHO
3	Chidubem Ogbudebe	Regional M&E Advisor, Lagos	KNCV/CTB
4	Dr Austin Ihesie	Program Officer	KNCV/CTB
5	Dr Titilope Ogunlade	Program Officer -Benue	KNCV/CTB
6	Dr Nkem Chukwuemeka	Senior Program Manager. Lagos Region	KNCV/CTB
7	Abdul Rasak Dikko	Program Officer	KNCV/CTB
9	Babagana Adams	Manager	TB Network/Civil Society
10	Mayowa Joel		Africa Development/ Civil Society
11	Dr Osakwe		
12	Mrs Ibiyemi Fakande		Living Hope Care/Civil society

	Cecilia	Kafran	APFRAM
	Jenna		STOP TB
	Aisha		TB Network
13	Dr Emperor Ubochioma	Community Focal Lead	NTP
14	Dr Aboki Danjuma	STBLCO	Nasarawa State
15	Dr Festus Olukayode Soyinka	STBLCO	Ogun State
16	Gideon Zephaniah	Regional M&E Advisor, Kano	KNCV/CTB
17	Stephanie Gande	Regional M&E Advisor, Abuja Region	KNCV/CTB
18	ifiok Ekanim (Austin)	Regional M&E Advisor, Akwa Ibom Region	KNCV/CTB
19	Rupert Enogu	Senior TB/HIV Technical Advisor	KNCV/CTB
