# Supplementary material for: Iterative evaluation of mobile computer-assisted digital chest x-ray screening for TB improves efficiency, yield, and outcomes in Nigeria
Source: PLOS Glob Public Health. 2024 Jan 17;4(1):e0002018. doi: 10.1371/journal.pgph.0002018 (PMC10793917; doi:10.1371/journal.pgph.0002018)
Supplement: S2 File — (PDF) [file pgph.0002018.s002.pdf]

Monitoring and Evaluation plan for WoW TB Campaign

The cascade of care

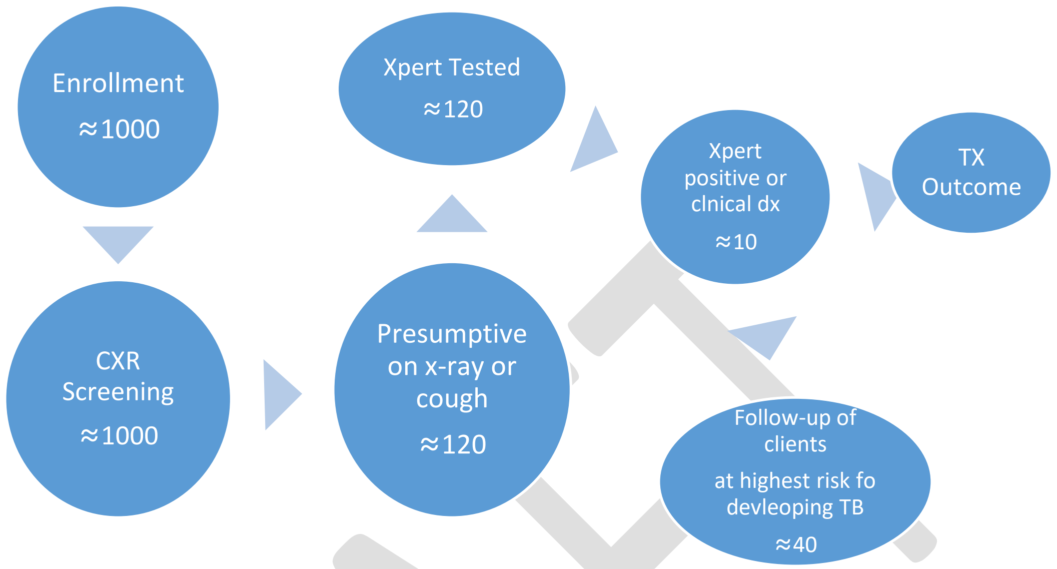

Figure 1: Cascade of care for the WoW TB Screening campaign

Cascade of care (Enrollment → Screening → Diagnosis → Treatment → Treatment outcome) → and re-testing of those at highest risk for progression to active TB.

Key performance indicators

The indicators will monitor the cascade of care. These will be used for quality and performance improvement as well. The indictors include:

A. CORE INDICATORS

1. Number of enrollees by age, sex and cough status
2. Number of enrollees screened by chest x-ray by age, sex and cough status
3. Number of presumptive TB clients (by cough symptomatic, CXR and both)
4. Number of presumptive TB clients investigated for TB by age and sex
5. Number diagnosed with TB by age, sex, type of TB (DS-TB and RR-TB), cough status and CAD4TB score
6. Number of presumptive TB clients eligible for follow up testing (i.e. CAD4TB score > 80 and MTB not detected on GeneXpert) by age and sex
7. Number of presumptive TB clients eligible for follow up testing enrolled on "TB diagnosis follow up program".
8. Number of newly diagnosed TB patients, whose contacts were screened
9. Number of household contacts of newly diagnosed TB patients by age and sex
10. Number of household contacts who are screened for TB by age and sex

11. Number of household contacts who become presumptive TB clients by age and sex
12. Number of household contacts who are presumptive TB clients tested for TB by age and sex
13. Number of household contacts who are diagnosed with TB by age and sex

B. QUALITY INDICATORS

1. Number of enrollees not screened by chest x-ray
2. Number of presumptive TB clients not investigated for TB by age and sex
3. Number of TB patients diagnosed with TB who received their GXP results
4. Number of TB patients who started TB treatment (by type of treatment – 1st line and 2nd line treatment)
5. Number of DS-TB patients who did not start treatment 2 weeks after diagnosis (pre-treatment Loss to follow up)
6. Number of RR-TB patients who did not start treatment 1 month after diagnosis (pre-treatment Loss to follow up)
7. TB patients administered with the health seeking behaviour and risk factors questionnaire
8. TB patients with documented HIV status
9. HIV-infected TB patients on ART during TB treatment
10. HIV-infected TB patients on CPT during TB treatment
11. TB Treatment outcome using standard cohort analyses
12. Number of household contacts diagnosed with TB started on treatment by type of TB (DS-TB and DR-TB)
13. Number of household contacts eligible for IPT by age and sex
14. Number of household contacts eligible for IPT started on IPT by age and sex

Tools for data collection and reporting

The recording and reporting tools that will be used for the intervention include:

1. WoW tally Card
2. Client Enrolment Tally Sheet
3. Client Enrolment Summary Form
4. Chest X-ray screening tally sheet
5. Chest X-ray screening summary form
6. Form for specimen Examination request
7. Clinic Register for Presumptive Drug Susceptible and Drug resistance TB Cases
8. Presumptive TB Clients summary form
9. GeneXpert test result Summary form
10. Line list for Diagnosed DS TB and DR TB patients
11. Line list for presumptive cases with undocumented test result
12. TB patient risk factors questionnaire
13. Client Follow up log sheet
14. Contact investigation tracking tool
15. CAD4TB database
16. GxAlert database

Guide for completing the tools

1. WoW Tally Card

Tally card (figure x) will be issued to clients as they enroll for the screening exercise.

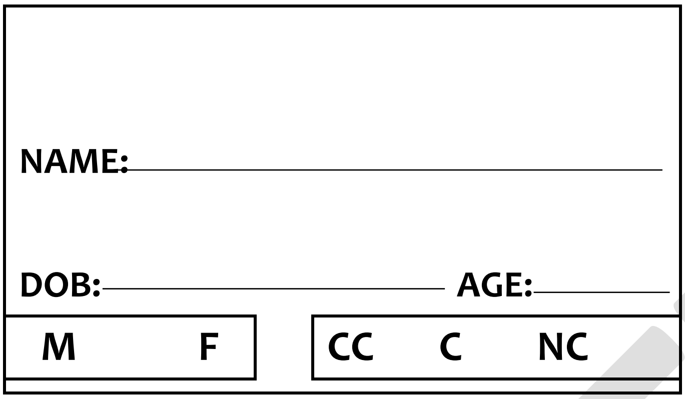

Figure 2: WoW tally card

The data clerk enrolling clients should complete the tally card as follows:

a. Serially print a barcode and stick to the upper part of the card
b. **Name:** Write the name of the client (Surname, First name and second name)
c. **DOB:** Write the date of birth of the client. Estimate if not known. If client is born in the first half of a particular year, use March 31 as the birthday, and September 30 as the birthday; if the second half of the year
d. **Age:** Calcualte the age and enter the value
e. **Sex:** Circle "M" if client is a male and "F" if client is a female.
f. **Cough Status: –** Circle CC, if client reports cough of 2 or more weeks; C, if client reports cough of less than 2 weeks and NC if cleint reports no cough.

2. Patient Barcode Format Guide

➢ PATIENT ID

There are two different unique Patient ID format for {Kano and Lagos WOW TRUCK}

Note: Both truck use the same **Order Naming Convention** but different **Unique Patient ID Numbering convention** Sequence. This is well explained in details below

❖ The barcode format have **10 Characters** which include the year, month, day and patient ID.
❖ The naming convention follows this order (Order Naming Convention)
YEAR+MONTH+DAY+PATIENT ID

Note 1: Two digits is use to represent the year, month and day

For example

- Year = 18 (i.e last two digits of the year)
- Month = 03 (i.e March)
- Day = 01 (i.e the first day)

DRAFT

Note 2: Four unique ID digits is use to identify the TB patient. Kano and Lagos WOW truck use different **Range of Numbering sequence**

- Kano = 0001 – 0999
- Lagos = 1001 – 1999
    - 1803010001 (**Kano first Patient ID in the month of March**)
    - 1803011001 (**Lagos first Patient ID in the month of March**)

BARCODE PREVIEW

WoW Client ID
1803010001

Kano first patient ID View

WoW Client ID
1803011001

Lagos first Patient ID view

- ID for Household contacts of an Index TB PATIENT

Contact investigation will be carried out for all diagnosed TB patients. Contacts who are presumed to have TB (presumptive Tb clients) will be issued a unique barcode number that will link them to their index patient.

The barcode number for each contact who is a presumptive case will be a 12 digit number with the first 10 identical to his/her index patient, while the last is of the numbering sequence 01 - 99

Example: if there are three presumptive clients among the contacts of a particular index case (with barcode number: **1803010001**) the barcode numbers of the presumptive clients among the contacts will be:

- First contact person ------------------ 180301000101
- Second contact person--------------180301000102

DRAFT

- Third contact person------------------180301000103

Each contact has a 12 digit ID. The first 10 are identical for all contacts, because they are contacts of the same person, while the last two digits (contact ID) differ and represent the serial number of the contacts

WoW Client ID

180301000101

BARCODE NUMBER OF THE FIRST CONTACT OF AN INDEX PATIENT

3. *Client Enrolment Tally Sheet*

The data clerk who issues **tally card** to clients will complete the client enrolment tally sheet as follows:

a. Fill the top half of the sheet:
   - **State:** Write the name of the state where screening is taking place.
   - **LGA:** Write the name of LGA where screening is taking place.
   - **Ward code:** Enter the code of the ward (see list) where screening is taking place.
   - **Location:** Indicate the location (name of the site) where screening is taking place.
   - **Geocodes:**
     - Using a geocordinate reader, enter the latitude and longitude of the screening site in in Degrees, minutes, and seconds. *e.g. Latitide: 9° 2' 23.85" N and longitude: 7° 28' 4.28" E*

b. Using tally system, indicate in the appropraite cells the age group, gender and cough status of all clients issued with tally cards.

4. *Client Enrolment Summary Form*

The client enrolment summary form provides information on client enrolment disaggregated by gender, age group and cough status. The purpose of the form is to ensure that the right mix of age and gender of clients are enrolled. It should be completed weekly and/or at the end of screening in a particular location.

The data clerk should complete the form as follows:

- **State:** Write the name of the state where screening is taking place.
- **LGA:** Write the name of LGA where screening is taking place.
- **Ward code:** Enter the code of the ward (see list) where screening is taking place.
- **Location:** Indicate the location (name of the site) where screening is taking place.

DRAFT

- Geocodes:
    - Using a geocordinate reader, enter the latitude and longitude of the screening site in in Degrees, minutes, and seconds *e.g. Latitide: 9° 2’ 23.85” N and longitude: 7° 28’ 4.28” E*
- **Age, Gender and Cough Status:** Using the enrollment tally sheet, enter the number of clients as appropriate for the age group, gender and cough status as shown in the sheet.
- **Total:** Sum of all clients in each row and column as appropriate
- **Name and signature:** Full names and signature of the WOW project staff who filled the weekly summary report.
- **Period:** Indicate the period of report e.g. from 02/04/2018 to 06/04/2018
- **No of screening days:** Indicate the number of days screening was conducted during the reporting period.

Note: This summary form should be completed using the Daily Client Enrolment Tally Sheet

5. Chest X-ray Screening Tally Sheet

The radiographer or assistant completes this tally sheet as follows:

c. Fill the top half of the sheet:
- **State:** Write the name of the state where screening is taking place.
- **LGA:** Write the name of LGA where screening is taking place.
- **Ward code:** Enter the code of the ward (see list) where screening is taking place.
- **Location:** Indicate the location (name of the site) where screening is taking place.
- **Geocodes:**
    - Using a geocordinate reader, enter the latitude and longitude of the screening site in in Degrees, minutes, and seconds. *e.g. Latitide: 9° 2’ 23.85” N and longitude: 7° 28’ 4.28” E*

d. Using tally system, indicate in the appropraite cells the age group, gender and cough status of all clients to be x-rayed.

6. Chest X-ray Screening Summary Form

The chest x-ray (CXR) screening summary form provides information on client’s screened with CXR disaggregated by gender, age and cough status. The purpose of the form is to track the number, age and sex of clients screened with CXR. It is used to ensure that the weekly screening target of 1000 is met and that the right mix of age and gender of clients is maintained. Nigerian men have twice the rate of bacteriologically confirmed pulmonary TB as women and they should represent 60 to 75% of the screened clients in the WoW project. It should be completed weekly and/or at the end of screening in a particular location.

The form is completed as follows:

- **State:** Write the name of the state where screening is taking place.
- **LGA:** Write the name of LGA where screening is taking place.

DRAFT

- **Ward code:** Enter the code of the ward (see list) where screening is taking place.
- **Location:** Indicate the location (name of the site) where screening is taking place.
- **Geocodes:**
    - Using a geocordinate reader, enter the latitude and longitude of the screening site in in Degrees, minutes, and seconds. *e.g. Latitide: 9° 2' 23.85" N and longitude: 7° 28' 4.28" E*
- **Age, Gender and Cough Status:** Using the screening tally sheet, enter the number of clients as appropriate for the age group, gender and cough status as shown in the sheet.
- **Total:** Sum of all clients in each row and column as appropriate
- **Name and signature:** Full names and signature of the WOW project staff who filled the weekly summary report.
- **Period:** Indicate the period of report e.g. from 02/04/2018 to 06/04/2018
- **No of screening days:** Indicate the number of days screening was conducted during the reporting period.

Note: This summary form should be completed using the chest x-ray screening tally sheet

7. Clinic Register for Presumptive Drug Susceptible and Drug resistance TB Clients

This register should be filled whenever a presumptive TB client is identified, when the sputum specimen is collected for diagnosis and later when the health worker receives the GeneXpert result as follows:

Sputum Identification Number: this is the registration number of the sputum specimen at time of collection. The number will be derived as follows: Truck code/Number assigned to presumptive TB case in the month/ month/ number of specimen. For example the 1st sputum specimen that was taken from a presumptive TB client at WoW (Southern region) in July, being the 64th client that was screened that month, would be labelled: WoW 1/64/07/1. Please note the two WoW trucks are coded as follows: WoW 1, for the truck in the southern region and WoW 2, for the truck in the northern region

Full Name: Write the client's full name and phone number.
Age: Age of client, estimate, if not known.
Gender: Write letter "M" if client is a male and letter "F" if client is a female.
Hospital/Clinic No.: Write the unique ID number on the barcode.
Full Address: The client's full address (traceable address) where client could be found if he/she does not return to commence treatment.
Source of referral: Write the Cad4TB score of the client in this column.
Specify reason for test (if test requested is GeneXpert MTB/Rif): Indicate the cough status of the client i.e. CC, C and NC
Date of collection: Write the date sputum sample was collected in this column.
Date sputum result released: The date the results of the GeneXpert was released is written in this column
AFB Result: Not Applicable.
Known HIV status: write letter Y if HIV status is known and N if not known at the moment.

DRAFT

HIV on ART: Indicate by ticking if patient is on ART.
Counselled: write Y if counselled and N if patient declined.
HIV tested: write Y if tested and N if not tested
HIV Result: Tick as appropriate.
LGA TB Registration Number: Immediately the sputum result is out, register this patient for treatment and complete TB treatment card. The LGTBLS will assign LGTBL number subsequently that should be documented on the register.

8. Presumptive TB Clients summary form

The presumptive TB Clients summary form must be filled at the end of every week’s activity or at the end of work in a particular location. The data clerk should complete the tool as follows:

- **State:** Write the name of the state where screening is taking place.
- **LGA:** Write the name of LGA where screening is taking place.
- **Ward code:** Enter the code of the ward (see list) where screening is taking place.
- **Location:** Indicate the location (name of the site) where screening is taking place.
- **Geocodes:**
  - Using a geocordinate reader, enter the latitude and longitude of the screening site in in Degrees, minutes, and seconds. *e.g. Latitide: 9° 2’ 23.85” N and longitude: 7° 28’ 4.28” E*
- **Age Group:** Enter the number of clients as appropriate for the age group provided in the sheet.
- **Gender:** Enter the number of the following by gender i.e. male or female (X-ray normal + cough≥ 2weeks, X-ray abnormal + cough ≥ 2weeks, X-ray abnormal + cough <2weeks, X-ray abnormal + no cough and the total in each row).
- **X-ray normal + cough≥ 2 weeks:** Enter the number of clients identified as presumptive TB clients who have normal X-ray result, but have a cough of ≥ 2 weeks duration as appropriate in the boxes disaggregated by gender and age group.
- **X-ray abnormal + cough ≥ 2 weeks:** Enter the number of clients identified as presumptive TB clients who have abnormal X-ray result and have a cough of ≥ 2 weeks duration as appropriate in the boxes disaggregated by gender and age group.
- **X-ray abnormal + cough <2weeks:** Enter the number of clients identified as presumptive TB clients who have abnormal X-ray result and coughing for less than 2 weeks as appropriate in the boxes disaggregated by gender and age group.
- **X-ray abnormal + no cough:** Enter the number of clients identified as presumptive TB clients who have abnormal X-ray result and not coughing as appropriate in the boxes disaggregated by gender and age group.
- **Total:** Sum of all clients in each row and column as appropriate.
- **Name of Responsible person:** Full names of the WOW project staff who filled the weekly summary report.
- **Signature:** The WOW project staff filling the summary report should sign in this space.
- **Date:** Date (day/month/year) the form was filled.

Note: This form should be completed using the presumptive TB case register

DRAFT

9. *Presumptive TB Clients' GeneXpert test result Summary form*

This form provides information on presumptive TB clients GeneXpert test result disaggregated by gender, age group, X-ray result and cough status, and must be completed at the end of every week's activity. The healthcare worker should complete the form as follows:

- **State:** Write the name of the state where screening is taking place.
- **LGA:** Write the name of LGA where screening is taking place.
- **Ward code:** Enter the code of the ward (see list) where screening is taking place.
- **Location:** Indicate the location (name of the site) where screening is taking place.
- **Geocodes:**
    - Using a geocordinate reader, enter the latitude and longitude of the screening site in in Degrees, minutes, and seconds. *e.g. Latitide: 9° 2' 23.85" N and longitude: 7° 28' 4.28" E*

- **Age Group:** Enter the number of clients as appropriate for the age group provided in the sheet.
- **Gender:** Enter the number of the following by gender i.e. male or female (MTB not detected, MTB Detected Rif not detected, MTB detected Rif resistant detected and MTB detected Rif resistant detected) as appropriate.
- **Successful test results:** GeneXpert test results recorded as MTB not detected, MTB Detected Rif not detected, MTB detected Rif resistant detected and MTB detected Rif resistant detected.
- **X-ray normal + cough≥ 2 weeks:** Enter the number of clients identified as presumptive TB who have normal X-ray result and coughing for ≥ 2 weeks as appropriate in the boxes disaggregated by gender, age group and GeneXpert test result.
- **X-ray abnormal + cough ≥ 2 weeks:** Enter the number of clients identified as presumptive TB who have abnormal X-ray result and coughing for ≥ 2 weeks as appropriate in the boxes disaggregated by gender, age group and GeneXpert test result.
- **X-ray abnormal + cough < 2weeks:** Enter the number of clients identified as presumptive TB who have abnormal X-ray result and coughing for less than 2 weeks as appropriate in the boxes disaggregated by gender, age group and GeneXpert test result.
- **X-ray abnormal + no cough:** Enter the number of clients identified as presumptive TB who have abnormal X-ray result and not coughing as appropriate in the boxes disaggregated by gender, age group and GeneXpert test result.
- **Total:** Sum of all clients in each row and column as appropriate.
- **Name and signature:** Full names and signature of the WOW project staff who filled the weekly summary report.
- **Period:** Indicate the period of report e.g. from 02/04/2018 to 06/04/2018
- **No of days GeneXpert test was conducted:** Indicate the number of days screening was conducted during the reporting period.

Note: This form should be completed using the presumptive TB case register

DRAFT

10. Line list for Diagnosed DS TB and DR TB patients

This is a quality care tool. It documents the care package for each newly diagnosed TB patient. The ACF coordinator should complete the line list of diagnosed TB cases as follows:

- **Unique ID Number:** Write the unique barcode number of the patient.
- **LGA TB Registration Number:** LGA TB Number is assigned to the patient at the time of registration in the LGA TB Central Register. This information will be completed only after the patient is registered in the LGA Tuberculosis Central Register by the LGATBLS.
- **Patient's Name:** Write the patient's full name.
- **Gender:** Tick "M" box if the patient is a Male and "F" if patient is a Female.
- **Age:** Write the age (in years) of the patient. If the patient does not know his/her age, make an estimation.
- **Patient's Phone number:** Write phone number of patient, if not available obtain phone number of the contact person or treatment supporter.
- **Type of TB (DS TB/DR TB):** Indicate as appropriate the type of patient: DS-TB (drug susceptible TB) or DR-TB (drug-resistance TB).
- **GeneXpert test result:** Indicate the GeneXpert test results with the following codes:
    - a = MTB Not Detected
    - b = MTB Detected RIF Resistance Not detected
    - c = MTB Detected RIF Resistance Detected
    - d = MTB Detected RIF Resistance Indeterminate
    - e = error
    - f= Invalid
    - g = Incomplete
- **Date diagnosis was made:** Write the day/month/year TB diagnosis was made i.e. date of GeneXpert test result was released or date of confirmation of clinical diagnosis.
- **Date Patient received GeneXpert result:** Write the day/month/year TB patient recieved the GeneXpert test result
- **Date treatment started:** Write the day/month/year patient started TB treatment in this space.
- **Duration (days) between diagnosis and treatment enrolment:** Indicate the interval (days) between patient's date (day/month/year) of diagnosis and date (day/month/year) treatment started.
- **Date questionnaire was administered:** Write the day/month/year the questionnaire was administered to the newly diagnosed TB patient.
- **Where patient is enrolled on treatment:** Indicate the facility, LGA and state where the patient is enrolled on treatment
- **HIV-infected TB patients on ART during TB treatment:** Indicate yes or no as appropriate
- **HIV-infected TB patients on CPT during TB treatment:** Indicate yes or no as appropriate
- **Treatment Outcome:** Write the appropriate treatment outcome, e.g. cured, treatment completed etc.
- **Remarks:** Provide any comment about the patient in this space e.g. pregnancy, reasons for non-commencement or discontinuation of drug collection.

DRAFT

11. *Line list for presumptive cases with undocumented test result*

- **Name of client:** Write the name of the presumptive TB client
- **Date identified as presumptive TB case:** Write the date (day/month/year) the presumptive TB client was identified
- **Age:** Write the age of the client
- **Sex:** Write the gender of the client
- **CAD4TB score:** Write the CAD4TB score of the client
- **Cough status:** Indicate the cough status of the client i.e. CC, C and NC
- **Reason for undocumented test result:** select from the drop down the reason for the undocumented test

12. *Client Follow up log sheet*

This register must be filled for clients on follow up GeneXpert test, when the initial test result for diagnosis is obtained and later when the health worker receives the follow up GeneXpert result. The healthcare worker who collects information of clients with abnormal X-ray and MTB not detected GeneXpert result fills the register as follows:

- **Unique ID Number:** Write the unique barcode number of the patient.
- **Client's Name:** Write the client's full name.
- **Gender:** Tick "M" box if the client is a Male and "F" if patient is a Female.
- **Age:** Write the age (in years) of the patient. If the patient does not know his/her age, make estimation.
- **Client's Phone number:** Write phone number of client, if not available obtain phone number of the contact person or treatment supporter.
- **Full address of Client:** The client's full address (traceable address) where client could be found if he/she does not return to the health unit for repeat GeneXpert test.
- **Date of X-ray examination:** Record the date (day/month/year) x-ray was done at the truck site.
- **CAD4TB Score:** Write client's CAD4TB score.
- **GeneXpert test result:** Indicate the GeneXpert test results with the following codes:
    - a = MTB Not Detected
    - b = MTB Detected RIF Resistance Not detected
    - c = MTB Detected RIF Resistance Detected
    - d = MTB Detected RIF Resistance Indeterminate
    - e = error
    - f= Invalid
    - g = Incomplete
- **Date of GeneXpert test:** Write the day/month/year the client's GeneXpert result was released.
- **HIV Status:** Indicate "+" if the HIV result is reactive (positive) and "O" if HIV result is not reactive (negative) and " I " if indeterminate.
- **GeneXpert site for follow up tests:** Write the name of the GeneXpert site where the client will repeat GeneXpert test.

DRAFT

- **Text message reminder for follow-up GeneXpert test:** Write the date client will be contact to repeat GeneXpert test i.e. 3 months and 6 months after the initial GeneXpert test.
- **1st follow-up GeneXpert test:** Write the date (dd/mm/yyyy) GeneXpert test was repeated, and the GeneXpert test result as above
- **TB diagnosed:** Write 'Yes' or 'No' if TB was diagnosed.
- **Enrolled on treatment:** Write 'Yes' or 'No' if client was started on TB treatment (DS-TB or DR-TB).
- **Remarks:** Provide any comment about the patient in this space e.g. pregnancy, reasons for non-commencement or discontinuation of drug collection.

13. Contact Investigation Tracking tool

The tool is used to document the cascade of care provided to contacts of index TB patients diagnosed through the screening exercise.

Name of index cases: Write the name of the index TB patient

Unique ID Number: Write the unique ID number of the patient as generated by the barcode system

Date CI was done: write the date the contact investigation was carried out in the form dd/mm/yyyy. This also indicates that the contact investigation was done.

Number of household contacts: Write the number of household contacts (person who shared the same enclosed living space for one or more nights or for frequent or extended periods during the day with the index patient during the 3 months before the patient started the current treatment episode) of the index case.

Number of household contacts screened for TB: Write the number of household contacts who were screened (symptomatically and/or CXR) for TB

Number of presumptive TB clients identified: Write the number of presumptive TB clients identified through symptomatic and/or CXR screening

Number of presumptive TB clients tested for TB: Write the number of presumptive TB clients among screened contacts who were investigated for TB using GeneXpert

Number of TB patients diagnosed: Write number of presumptive TB clients among screened contacts diagnosed with TB (DS-TB or RR-TB)

Number of TB patients started on treatment: Write number of TB patients started on the appropriate treatment

Number of contacts eligible for IPT: Write number of contacts eligible (Under-six children and PLHIV without active TB) for isoniazid preventive therapy. *If an under-six is also a PLHIV, classify as PLHIV.*

Number of contacts eligible for IPT started on IPT: Write number of contacts eligible for IPT who were started on IPT.

DRAFT

14. CAD4TB DATABASE

- Open the CAD4TB data entry platform
- Use the barcode reader to scan the barcode on the client's tally card and automatically enter the client's unique ID number on the **patient ID text box**
- Enter the cough status of clients as it appears on the tally card by including a prefix (**CC, C** and **NC**) before the client's name.
- Enter the client's name (preceded by the cough status as prefix) in the **name text box** (in this order: Surname, First name and Middle Name),
- Enter the client's DOB in the **DOB text box**
- Enter the client's gender in the **gender text box**

CC	C	NC	CC	C	NC	CC	C	NC

- For instance, three clients with cough status as shown above will be documented as follows:
    - **CC** Surname Firstname Secondname
    - **C** Surname Firstname Secondname
    - **NC** Surname Firstname Secondname

15. On the GXAlert Software

- Click on the GxAlert data entry platform
- Use the barcode reader to scan the barcode on the client's sputum cup to automatically enter the client's ID number on the **patient ID text box**. In the same field, leave a space and write the client's name.
- Enter the client's name in the **name text box** (in this order: Surname, First name and Middle Name),
- Enter the client's age in the **age text box**
- Enter the client's gender in the **gender text box**
- Enter the client's cough in the **cough text box**
- Select client's state from the drop down
- Select client's LGA from the drop down
- Enter code for client's risk group in the text box for "**Name of Person Requesting Test**" (Prison = 1, HIV clinic = 2, Urban slum = 3, Household contact = 4, OPD = 5, Others = 6)
- Enter the **ward code of the test site** (see list) in the **Health Facility text box**.
- Enter the cough status of the client in the **referring clinician/phone number text box** (cough+ = cough of 2 or more weeks and cough- = cough of less than 2 weeks or no cough).

16. TB patient risk factors and KAP questionnaire

- For each diagnosed TB patient, print a third barcode (with same number) and stick on the questionnaire
- Administer the questionnaire following the instructions contained in the questionnaire
- Keep the completed quesionnaire in a file and store away

The ACF coordinator should submit the following summary tools weekly:

DRAFT

1. Client Enrolment Summary Form
2. Chest X-ray screening summary form
3. Presumptive TB Clients summary form
4. GeneXpert test result Summary form
5. Line list for Diagnosed DS TB and DR TB patients
6. Line list for presumptive cases with undocumented test result
7. GeneXpert database download
8. CAD4TB database download

The ACF coordinator should submit the following summary tools monthly:

9. Contact investigation tracking tool
10. Client Follow up log sheet

At the end of work in each cluster, the ACF coordinator shall complete a field summary report that includes information on the indicators below:

This information is necessary to provide timely feedback to implementers as well as LGA and facility staff. All reports should be sent to central level for data entry and analysis.

DRAFT

M&E Framework

Indicators	Definition of indicators	Baseline	Target	Data source	Frequency	Responsibility
CXR screening rate	(Number of enrollees screened by CXR over a period of time divided by number of persons enrolled for screening over same period) %	NA	90%	Client Enrolment Summary Form and CXR screening Summary Form	Weekly	Data clerk
GeneXpert investigation rate	(Number of presumptive TB clients investigated for TB by GeneXpert assay divided by number of presumptive TB clients) %	NA	≥ 95%	Merged CAD4TB and GeneXpert database		Data clerk
GeneXpert result retrieval rate	(Number TB patients who received their GXP results divided by number of TB patients) %	NA	<1%	Line list of diagnosed patients		
MTB positivity rate	(Number of people diagnosed with any form of TB divided by number of presumptive TB clients investigated for TB) %	NA	10%	GeneXpert test result Summary form or Merged CAD4TB and GeneXpert database		Data clerk
Rifampicin resistance rate among all TB patients	(Number of people diagnosed with any form of TB divided by number of presumptive TB clients investigated for TB) %	NA	2%	GeneXpert test result Summary form or Merged CAD4TB and GeneXpert database		Data clerk
Initial loss to follow up rate among DS-TB patients	Number of people diagnosed with DS-TB who did not start treatment with 1st line anti-TB medicines within 2 weeks after diagnosis	NA	< 5%	Line list for Diagnosed DS TB and DR TB patients and case		ACF Coordinator

	divided by number of people diagnosed with DS-TB expressed as a percentage			finding summary form		
Initial loss to follow up rate among RR-TB patients	Number of people diagnosed with RR-TB who did not start appropriate treatment 1 month after diagnosis divided by number of people diagnosed with RR-TB expressed as a percentage	NA	< 5%	Line list for Diagnosed DS TB and DR TB patients and case finding summary form		ACF Coordinator
Enrollment rate of DS-TB patients on 1st line anti-TB medicines	(Number of people diagnosed with DS-TB who started 1st line anti-TB medicine divided by number of people diagnosed with DS-TB) %	NA	> 98%	Line listing		
Treatment success rate among patients on 1st line anti-TB medicine	(Number of DS-TB patients who were successful (cured and completed) with treatment divided by the number of DS-TB patients started on treatment over a specified period of time) %	NA	> 90%	Cohort report		ACF Coordinator
Lost to follow up rate among patients on 1st line anti-TB medicine	(Number of DS-TB patients started on 1st line anti-TB medicines but interrupted treatment for ≥ 2 months divided by the number of DS-TB patients started on treatment over a specified period of time) %	NA	< 4%	Cohort report		ACF Coordinator
Failure rate among patients on 1st line anti-TB medicine	(Number of DS-TB patients on 1st line anti-TB medicine whose sputum smear or culture is positive at month 5 or later during	NA	< 3%	Cohort report		ACF Coordinator

DRAFT

	treatment divided by the number of DS-TB patients started on treatment over a specified period of time) %					
Death rate among patients on 1st line anti-TB medicine	(Number of DS-TB patient on 1st line anti-TB medicine who died in the course of treatment divided by the number of DS-TB patients started on treatment over a specified period of time) %	NA	< 3%	Cohort report		ACF Coordinator
Enrollment rate of RR-TB patients on 2nd line anti-TB medicines	(Number of people diagnosed with RR-TB who started 2nd line anti-TB medicine divided by number of people diagnosed with RR-TB) %	NA	> 95%	Line listing		ACF Coordinator
Enrollment rate for "TB diagnosis follow up program"	(Number of presumptive TB clients eligible for follow up diagnosis enrolled on "TB diagnosis follow up program" divided by number of presumptive TB clients eligible for follow up diagnosis) %	NA	100%	Client Follow up log sheet		ACF Coordinator
Percentage of registered TB patients with documented HIV status	(Number of registered TB patients with documented HIV status divided by number of registered TB patients) %	NA	95%	Line list of diagnosed patients		ACF Coordinator
Percentage of HIV-infected registered TB patients on ART during TB treatment	(Number of HIV-infected TB patients on ART during treatment divided by number of HIV-infected TB patients) %	NA	95%	Line list of diagnosed patients		ACF Coordinator

DRAFT

Percentage of HIV-infected registered TB patients on CPT during TB treatment	(Number of HIV-infected TB patients on CPT during treatment divided by number of HIV-infected TB patients) %	NA	95%	Line list of diagnosed patients		ACF Coordinator
Index patient contact investigation coverage	(Number of index patients for whom contacts are investigated divided by the total number of index patients) %	NA	90%	Contact investigation tracking tool		ACF Coordinator
Contact Investigation coverage	(Number of contacts who are investigated divided by the total number of identified contacts)%	NA	95%	Contact investigation tracking tool		ACF Coordinator
Investigation rate among household contacts who are presumptive TB clients	(Number of presumptive TB clients among contacts investigated for TB divided by number of presumptive TB clients among contacts) %	NA	>95%	Contact investigation tracking tool		ACF Coordinator
Prevalence of TB among screened household contacts	(Number of TB patients identified among screened contacts divided by number of screened contacts) %	NA	3%	Contact investigation tracking tool		ACF Coordinator
IPT enrollment rate among eligible contacts	(Number of contacts eligible for IPT placed on IPT divided by eligible contacts) %	NA	65%	Contact investigation tracking tool		ACF Coordinator
Number Needed to Screen (NNS) to find 1 true case – (same-day diagnosis)	Inverse of the prevalence of TB among those screened (same day diagnosis)	NA	100	Adapted presumptive TB register and Client Enrolment Summary Form		Data clerk
Number Needed to Screen (NNS) to find 1 true case – (follow up diagnosis)	Inverse of the prevalence of TB among those screened (follow up enrollees)	NA	TBD	Adapted presumptive TB register and Client		Data clerk

DRAFT

				Enrolment Summary Form		
Number Needed to Screen (NNS) to find 1 True case – (same-day and follow up diagnosis)	Inverse of the prevalence of TB among those screened (same day diagnosis and follow up enrollees)	NA	TBD	Adapted presumptive TB register and Client Enrolment Summary Form		Data clerk
