# Supplementary material for: Iterative evaluation of mobile computer-assisted digital chest x-ray screening for TB improves efficiency, yield, and outcomes in Nigeria
Source: PLOS Glob Public Health. 2024 Jan 17;4(1):e0002018. doi: 10.1371/journal.pgph.0002018 (PMC10793917; doi:10.1371/journal.pgph.0002018)
Supplement: S3 File — (PDF) [file pgph.0002018.s003.pdf]

Supplementary 3 data

S3 Figure A: Venn diagram of the relative contribution of CAD4TB and Cough≥ two weeks to B+TB case finding in the Calibration and Pilot phases

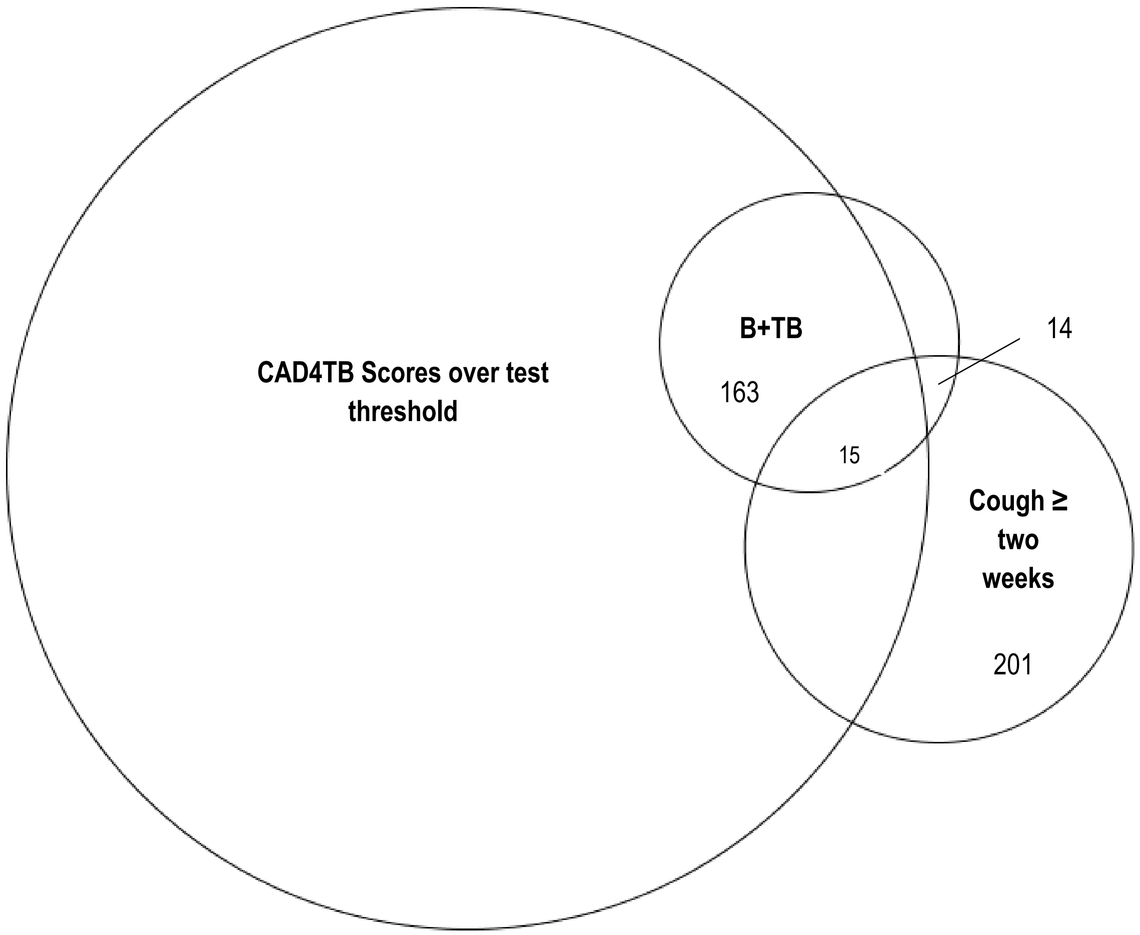

As the calibration and pilots did not include a reference test for all participants (but rather only 41% in the calibration, and % in the), these analyses are not true reflections of the accuracy of CAD4TB version 4 in this population. The ROC curve underestimates the sensitivity gradient below the test threshold. Nevertheless we made an indicative ROC curve and table in order to estimate the costs and benefits of lowering the testing threshold. The plot appeared to suggest that a threshold of 57 would give a gain of at least 1.4% with a cost of approximately 3% additional testing. On this basis, ≥57 was implemented as the testing threshold in the Northern truck where the test eligible proportion was low at 10.8% and could absorb additional presumptive clients.

The pseudo ROC curve and cost estimates were generated using an online tool developed by Takuya Yamanaka. The tool is available here: https://worldhealthorg.shinyapps.io/CAD4TB_validity/

Figure B: Pseudo receiver operating characteristic curve for CAD4TB v.4 during Calibration and Piloting

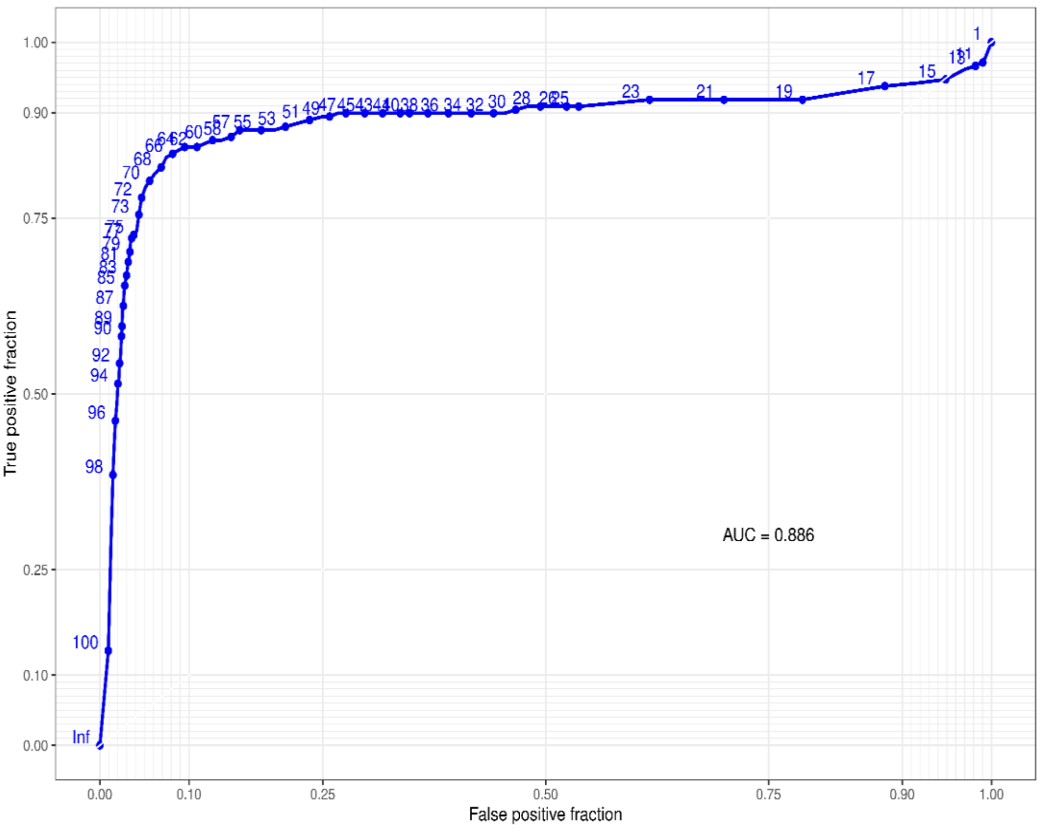

Table A: Estimated metrics for Algorithm optimization from Calibration and Pilot Phases

CAD4TB v.4 score testing threshold	Censored Sensitivity	Censored Specificity	Proportion needing a GeneXpert Test
48	89.90%	72.40%	28.50%
50	89.40%	74.10%	26.90%
51	88.90%	75.60%	25.40%
52	88.50%	77.00%	24.10%
53	88.00%	78.30%	22.70%
57	87.50%	83.40%	17.70%
58	86.50%	84.40%	16.70%
60	86.10%	86.50%	14.70%
61	85.60%	87.40%	13.80%
64	85.10%	89.60%	11.60%
65	84.60%	90.30%	10.80%

The actual sensitivity gain in the Northern truck attributable to the lowered testing threshold of ≥57 CAD$TB score was approximately 2%, while the specificity loss was roughly 1% (Figure C).

Figure C: Quasi ROC curve of the North Truck in the Scale Up period

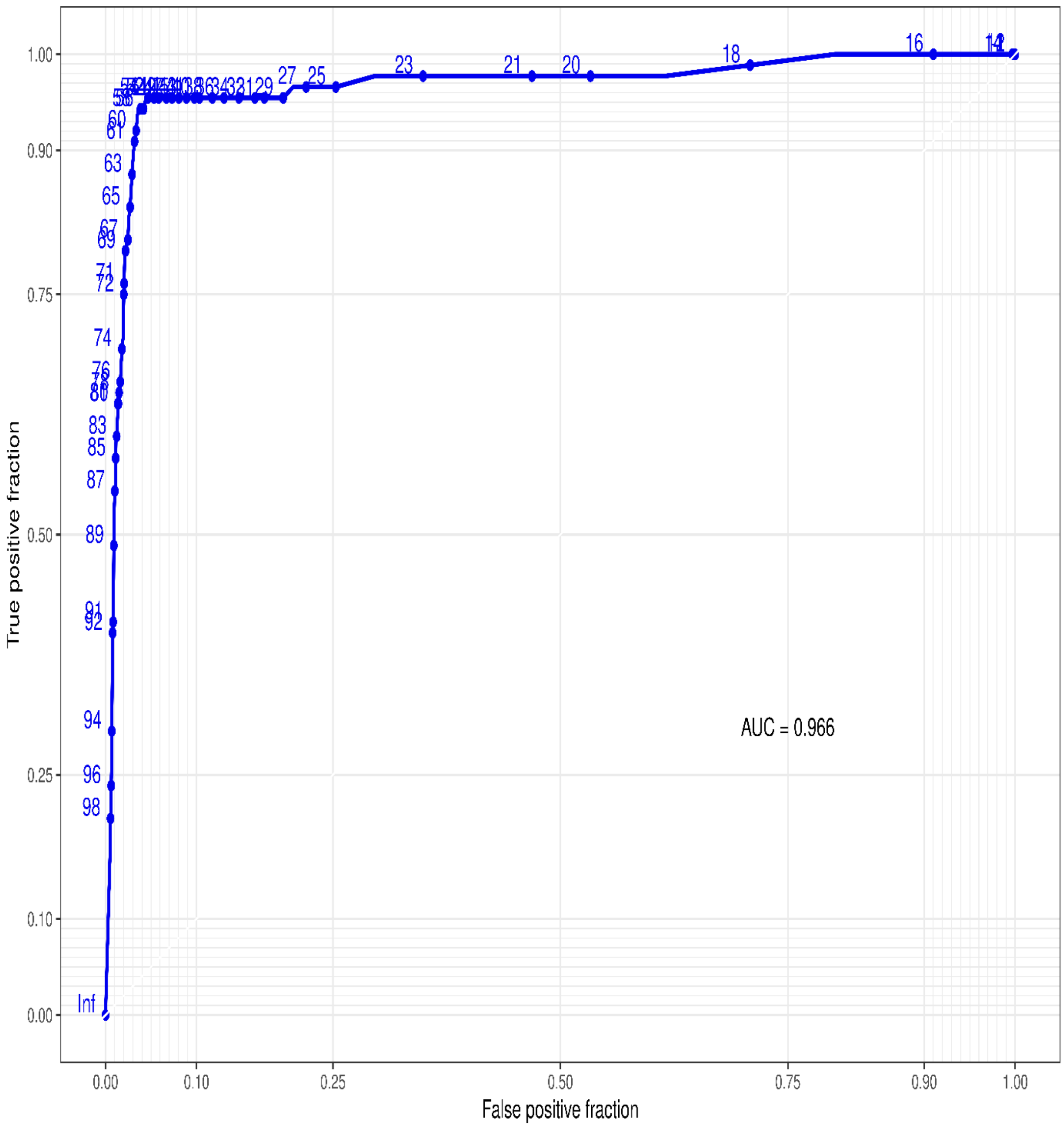

CAD4TB v.4 score testing threshold	Sensitivity	Specificity	Proportion needing a GeneXpert Test	Credibility
28	97%	79%	21%	Censored: sensitivity and specificity may be overestimated
55	96%	96%	5%	
59	94%	96%	4%	All received the diagnostic test
60	92%	97%	4%	
61	91%	97%	4%	
62	90%	97%	4%	

63	88%	97%	4%	
64	86%	97%	4%	
65	84%	97%	4%	
66	83%	97%	3%	

This ROC and accuracy table are censored, and may not reflect of the true accuracy of the tools below the testing threshold of 57 because a TB test was not offered to all participants, rather only those with chronic cough. Nevertheless the table do provide an indicative measure of the additionality of a ≥57 versus a ≥60 test threshold, as all participants in this category received the reference TB test. As a consequence of lowering the testing threshold to ≥57 in the North truck, two additional persons with TB were detected. Both had a CAD4TB score of 59, neither had symptoms.

Table B: Estimated metrics for Algorithm optimization from Calibration and Pilot Phase

CAD4TB score threshold	Proportion needing a GeneXpert Test	True Positives in 100,000 ppl	Missed cases (persons with TB CAD4TB score below threshold for testing)	False screen positives referred for testing	True Negatives screened negative	rough estimate of % of missed	Total cost for confirmatory test in USD	Cost per true case in USD	Extra cost due to referral to testing of true negatives in USD
48	29%	899	101	27622	724	10%	570,424	634	552,443
50	27%	894	106	25966	741	11%	537,195	601	519,310
51	25%	889	111	24518	756	11%	508,157	571	490,369
52	24%	885	115	23169	770	12%	481,066	544	463,373
53	23%	880	120	21856	783	12%	454,722	517	437,126
57	18%	875	125	16802	834	13%	353,540	404	336,040
58	17%	865	135	15846	844	14%	334,231	386	316,924
60	15%	861	139	13793	865	14%	293,070	341	275,858
61	14%	856	144	12900	874	14%	275,109	321	257,993
64	12%	851	149	10719	896	15%	231,402	272	214,383
65	11%	846	154	9998	903	15%	216,884	256	199,961

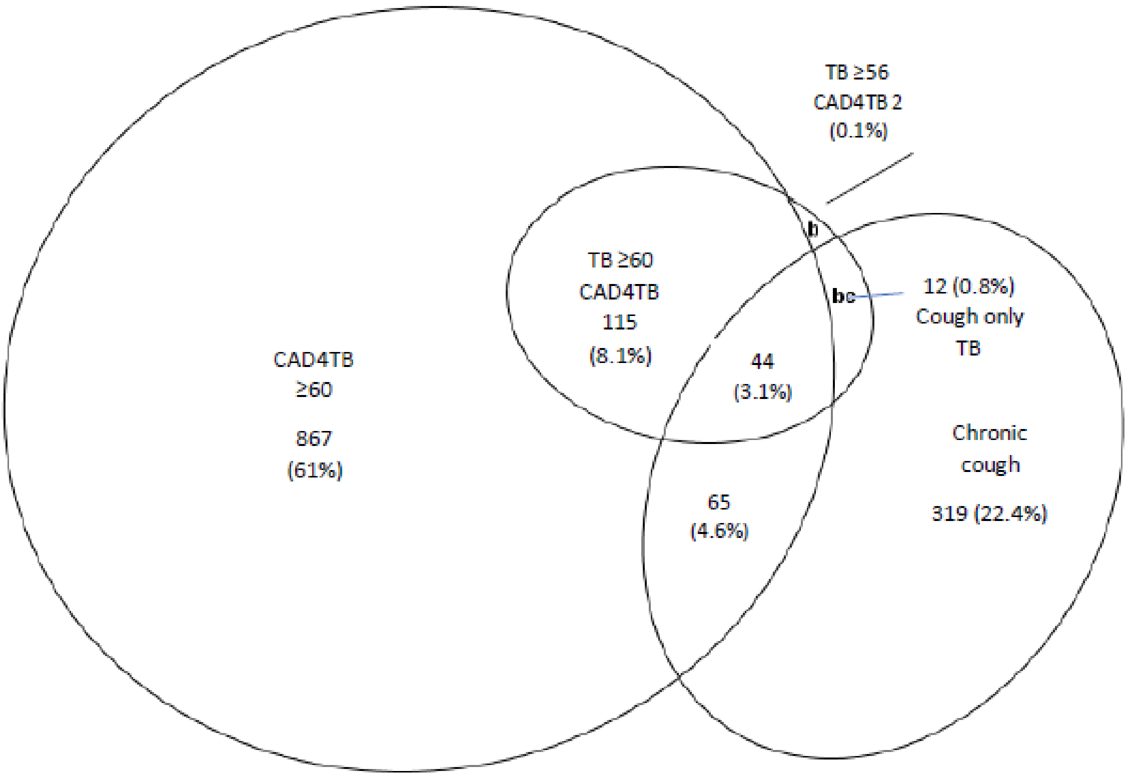
TB ≥56
CAD4TB 2
(0.1%)
TB ≥60
CAD4TB
115
(8.1%)
b
bc
12 (0.8%)
Cough only
TB
CAD4TB
≥60
867
(61%)
44
(3.1%)
Chronic
cough
65
(4.6%)
319 (22.4%)

Table E: Regional differences in Performance Metrics by Phase

Phase	Calibration		Pilot South		Pilot North		Pilot Total		Scale-Up South		Scale Up North		Scale-Up Total	
CAD4TB Test threshold	≥ 40		≥ 60 or <1		≥ 60 or <1		≥ 60 or <1		≥ 60 or <1		≥ 57 or <1		≥ 60 S and ≥ 57 N	
sample size	n=1,875		n=5,418		n=5,861		n=11,280		n=7,418		n=9,215		n=16,637	
Total days of screening	8		52		46		98		57		60		118	
**Acceptability in Population**	n	**%**	n	%	n	%	n	%	n	%	n	%	n	%
Average daily screen (SD)	203	**101**	129	65	127	64	128	**64.0**	122	61	154	77	140	**70.5**
Age over 30 years	1625	**87**	5135	95	3636	63	8771	**77.8**	5781	78	5599	61	11380	**68.4**
Male gender	721	**37**	2656	49	3896	67	6552	**58.1**	4155	56	6816	74	10971	**65.9**
Higher risk for TB	1751	**93**	5135	95	5181	90	10316	**91.5**	6945	94	8520	92	15465	**92.9**
Screened for chronic cough	1875	**100**	5418	100	5861	100	11279	**100.0**	7418	100	9215	100	16636	**100.0**
Screened with CXR	1865	**100**	5402	100	5835	100	11237	**99.6**	7396	100	9211	100	16615	**99.9**
Interpretable image	1844	**98**	5324	99	5795	99	11119	**98.9**	7325	99	9211	100	16540	**99.1**
Presumptive by chronic cough only	27	**1.4**	148	4	147	3	295	**2.6**	198	3	268	3	439	**2.6**
Presumptive by CAD4TB threshold	263	**41**	1133	21	409	7	1542	**13.7**	703	10	388	4	1091	**6.6**
Presumptive: eligible	769	**43**	1281	24	636	11	1917	**17.0**	880	12	647	7	1527	**10.6**
**Fidelity to Design and Protocol**														
Sample not produced, low quality, not tested	435	**56.5**	261	20	40	6	301	**15.7**	85	10	128	1	213	**13.9**
Tested for TB with GXP on-site among eligible	334	**43.**	1020	80	596	94	1616	**84.3**	795	90	519	80	1314	**86.1**
Bacteriologically confirmed TB	2	**0.001**	126	2	70	12	196	**1.7**	86	1	83	1	169	**1.0**
RIF resistant TB	1	**50**	1	1	3	4	4	**2.0**	7	8	0	0	7	**4.1**

Clinically diagnosed TB	0	0	0	0	0	0	0	0	1	1.1	2	2.4	3	**1.7**
Average daily yield	0.25		2.4		1.5		2.0000		1.5		1.4		1.46	
No. needed to screen (NNS) to detect 1 bacteriologically confirmed patient	958		42		84		58		85		108		98	
No. needed to test (NNT) detect 1 bacteriologically confirmed patient	385		7.9		8.5		8.2		9.2		6.1		7.6	
Cartridge costs per TB case (US$)	3842		78		85		82		92		62		76	
HIV tested	2	**100%**	111	97%	40	57%	151	**77.0%**	38	45%	75	90%	113	**65%**
TB Treatment initiation	2	**100%**	114	89%	56	80%	170	**87%**	83	97%	75	93%	158	**91%**
Treatment success	2	**100%**	96	76%	24	62%	120	**71%**	63	73%	71	88%	134	**85%**
