# Supplementary material for: Iterative evaluation of mobile computer-assisted digital chest x-ray screening for TB improves efficiency, yield, and outcomes in Nigeria
Source: PLOS Glob Public Health. 2024 Jan 17;4(1):e0002018. doi: 10.1371/journal.pgph.0002018 (PMC10793917; doi:10.1371/journal.pgph.0002018)
Supplement: Attachment — Submitted filename: RESPONSE TO REVIEWERS-updated.docx [file pgph.0002018.s004.docx]

| **Reviewer** | **Response** |
| --- | --- |
| 1. In the introduction, it will be useful to provide a few lines (2-3) on the implementation experience in other high TB burden countries, including its impact and feasibility | Additional studies have been referenced and the selective reporting of implementation experiences is addressed in the study rationale. |
| 2. Strengthen the rationale by explicitly stating the evidence gaps that this study will be fulfilling | OK, thanks, now re-phrased to make the study rationale more explicit. |
| 3. Describe the implementation settings eg health facilities, schools, factories and the rationale for their selection (if any) | See lines 173-175 on page 7: Community leaders, TB program staff, policy makers, and technical experts helped to select the locations for mobile outreach through a participatory mapping process (Table 1). This process is described in detail in S3 |
| 4. Discuss concordance/discordance of specific findings with other studies in the discussion section | We have expanded the discussion to highlight where our findings align or diverge from other studies. |
| 5. 1. You indicated that ethical approval was not necessary for your study. We understand that the framework for ethical oversight requirements for studies of this type may differ depending on the setting and we would appreciate some further clarification regarding your research. Could you please provide further details on why your study is exempt from the need for approval and confirmation from your institutional review board or research ethics committee (e.g., in the form of a letter or email correspondence) that ethics review was not necessary for this study? Please include a copy of the correspondence as an ""Other"" file. | We assumed that this study would have been exempted based upon an earlier exemption obtained from the Belgian IRB that was a secondary analysis of an overlapping dataset.  However, when we checked the Nigerian regulations, we realized after consulting with several Nigerian ethics stakeholders that we should not have assumed an exemption. We should have submitted it to the NHREC to seek exemption as well.  So we belatedly submitted a combined protocol of both studies using the overlapping secondary dataset to the NHREC in July, 2023 and the combined protocol was exempted from full review in October, 2023.  We attach the exemption letter. The names do to match because it is a combined protocol. We can submitt he combined protocol to verify this if that would be helpful. |
| 6. 2. Please send a completed 'Competing Interests' statement, including any COIs declared by your co-authors. If you have no competing interests to declare, please state "The authors have declared that no competing interests exist". Otherwise please declare all competing interests beginning with the statement "I have read the journal's policy and the authors of this manuscript have the following competing interests:" | Added and updated to reflect changing job of main author. |
| 7. 3. Please amend your detailed Financial Disclosure statement. This is published with the article. It must therefore be completed in full sentences and contain the exact wording you wish to be published.   a. State the initials, alongside each funding source, of each author to receive each grant. | added |
| 8. b. State what role the funders took in the study. If the funders had no role in your study, please state: “The funders had no role in study design, data collection and analysis, decision to publish, or preparation of the manuscript.” | added |
| 9. 4. Please note that your Data Availability Statement is currently missing  the DOI/accession number of each dataset OR a direct link to access each database. If your manuscript is accepted for publication, you will be asked to provide these details on a very short timeline. We therefore suggest that you provide this information now, though we will not hold up the peer review process if you are unable. | We have uploaded the data to dryad  Our submission has been assigned a unique digital object identifier (DOI): doi:10.5061/dryad.v9s4mw71p  According to Dryad policies, and our own requirements, we will not be able to include age, sex, HIV and treatment outcomes in case-based data with low level of geographic disaggregation due to risk of deductive disclosure.. |
| 10. 5. Please provide separate figure files in .tif or .eps format and remove the embedded figures from the manuscript file. | Done  DIVA-GIS (see reference below) is the source of the underlying layers and we have the permission to use this data for journals that use a CC-BY license.   - DIVA-GIS (2017) Free Spatial Data by Country. url: <http://www.diva-gis.org/gdata>   We have added the source to Figure 2 |
